# AIEgens for dark through-bond energy transfer: design, synthesis, theoretical study and application in ratiometric Hg^2+^ sensing†
†Electronic supplementary information (ESI) available: Synthetic procedures and characterizations, other experimental details. See DOI: 10.1039/c6sc04206f



**DOI:** 10.1039/c6sc04206f

**Published:** 2016-11-15

**Authors:** Yuncong Chen, Weijie Zhang, Yuanjing Cai, Ryan T. K. Kwok, Yubing Hu, Jacky W. Y. Lam, Xinggui Gu, Zikai He, Zheng Zhao, Xiaoyan Zheng, Bin Chen, Chen Gui, Ben Zhong Tang

**Affiliations:** a HKUST Shenzhen Research Institute , No. 9 Yuexing 1st RD, South Area, Hi-tech Park Nanshan , Shenzhen 518057 , China . Email: tangbenz@ust.hk; b Division of Biomedical Engineering , Department of Chemistry , Hong Kong Branch of Chinese National Engineering Research Center for Tissue Restoration and Reconstruction , Institute for Advanced Study , Institute of Molecular Functional Materials , State Key Laboratory of Molecular Neuroscience , The Hong Kong University of Science and Technology (HKUST) , Clear Water Bay , Kowloon , Hong Kong , China; c Guangdong Innovative Research Team , SCUT–HKUST Joint Research Laboratory , State Key Laboratory of Luminescent Materials and Devices , South China University of Technology (SCUT) , Guangzhou 510640 , China

## Abstract

A novel dark through-bond energy transfer (DTBET) strategy is proposed and applied as the design strategy to develop ratiometric Hg^2+^ sensors with high performance. Tetraphenylethene (**TPE**) derivatives with aggregation-induced emission (AIE) characteristics are selected as dark donors to eliminate emission leakage from the donors. The TBET mechanism has been adopted since it experiences less influence from spectral overlapping than Förster resonance energy transfer (FRET), making it more flexible for developing cassettes with large pseudo-Stokes shifts. In this work, energy transfer from the **TPE** derivatives (dark donor) to a rhodamine moiety (acceptor) was illustrated through photophysical spectroscopic studies and the energy transfer efficiency (ETE) was found to be up to 99%. In the solution state, no emission from the donors was observed and large pseudo-Stokes shifts were achieved (>280 nm), which are beneficial for biological imaging. Theoretical calculations were performed to gain a deeper mechanistic insight into the DTBET process and the structure–property relationship of the DTBET cassettes. Ratiometric Hg^2+^ sensors were rationally constructed based on the DTBET mechanism by taking advantage of the intense emission of **TPE** aggregates. The Hg^2+^ sensors exhibited well resolved emission peaks. >6000-fold ratiometric fluorescent enhancement is also achieved and the detection limit was found to be as low as 0.3 ppb. This newly proposed DTBET mechanism could be used to develop novel ratiometric sensors for various analytes and AIEgens with DTBET characteristics will have great potential in various areas including light harvesting materials, environmental science, chemical sensing, biological imaging and diagnostics.

## Abstract 2

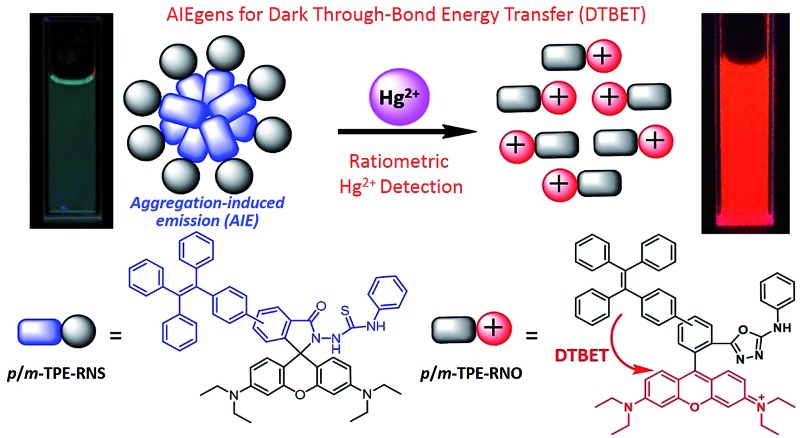
A novel dark through-bond energy transfer (DTBET) strategy is proposed and applied as the design strategy to develop ratiometric Hg^2+^ sensors with high performance.

## Introduction

Due to their high sensitivity, non-invasiveness and good spatial–temporal resolution, fluorescent techniques have fascinated scientists for decades in numerous applications such as chemical sensing, environmental science, biological imaging and medical diagnosis.^1–5^ Some frequently used emissive fluorogens such as BODIPY, fluorescein and rhodamine exhibit relatively small Stokes shifts (<30 nm), which brings some practical problems including the notorious inner filter effect and interference between the excitation and emission lights. Förster resonance energy transfer (FRET) is a powerful tool. It could provide a design strategy not only for developing fluorescent dyes with large pseudo-Stokes shifts but also for multicolour sensing and imaging.^6,7^ Normally, a FRET system is comprised of a donor and an acceptor which are connected by a flexible aliphatic spacer. The efficiency of the FRET is mainly tuned by alteration of three parameters: (1) the distance between the donor and the acceptor (*r*_DA_), (2) the degree of overlapping between the emission of the donor and the absorption of the acceptor (*J*), and (3) the relative orientation of the donor emission dipole moment and the acceptor absorption dipole moment.^1^ FRET has been widely used in many applications such as light harvesting in artificial photosynthesis and solar cells,^8–10^ chemical sensing,^11^ DNA and protein conformation change monitoring and enzyme activity detection.^12–16^

On the other hand, to achieve a large pseudo-Stokes shift, the spectral overlapping between donor emission and acceptor absorption might be diminished, which will lead to a decrement of the FRET efficiency and leakage of the donor emission. Burgess and co-workers have developed a new system with the mechanism of through-bond energy transfer (TBET), which could serve as a valuable approach to solve this paradox.^17,18^ In a TBET system, the donor and acceptor are connected by a rigid linker instead of a flexible aliphatic linker.^19–22^ It is noteworthy that the donor and acceptor are usually connected by a conjugated group (typically a phenyl ring, a double bond or a triple bond) and there is a large torsional angle between the donor and acceptor, preventing them from being treated as one fluorophore. The energy transfer rate in a TBET system can reach up to 2 orders of magnitude faster than that in a classical FRET system, making it less dependent on the spectral overlapping.^23–25^ Thus, one can easily achieve high energy transfer efficiency (ETE) through the TBET mechanism even if the spectral overlap is small, which is good for generating a large pseudo-Stokes shift.

Recently, Chang and co-workers proposed a novel FRET system called dark resonance energy transfer (DRET), which contains a dark donor with a low quantum yield (<1%).^26–28^ Fluorescent dyes with DRET demonstrate some attractive properties such as tuneable emission with a single excitation and a large pseudo-Stokes shift. Moreover, it is worth noting that there is no leakage from donor emission due to the low quantum yield of the donor, making the fluorescent dyes in the DRET library ideal candidates for biological applications. However, the ETEs of DRET dyes are still strongly dependent on spectral overlapping and the ETEs could be reduced when the non-radiative decay rate of the dark donor is fast enough to compete with the RET rate. As a result, the choice of donors and acceptors in DRET systems is limited. The introduction of the TBET mechanism to build a DTBET system could stand out as a more effective strategy. The TBET rate is more rapid relative to non-radiative decay and thus is less limited by overlapping of the spectra.^23^ Due to the low quantum yield of the donor, turn-on sensing can be easily realized by the dark energy transfer systems. However, fluorescent intensity is distinctly affected by the dye concentration, excitation power strength and other environmental factors, and it is hard for turn-on sensors to give quantitative information about the analytes. In this regard, ratiometric fluorescent probes are highly demanded for quantitative detection because they allow self-calibration at two wavelengths to eliminate most of the interference as mentioned above.^29^

To achieve ratiometric sensing abilities, aggregation-induced emission luminogens (AIEgens) are selected as the dark donor. AIEgens show no or very weak fluorescence in solution, but exhibit intense emission in the aggregated state, which is opposite to traditional dyes that usually show a notorious aggregation-caused quenching (ACQ) effect.^30^ The mechanism of the AIE is attributed to the restriction of intramolecular motion (RIM).^31^ Luminogens with AIE properties show superior features such as high brightness in the solid state and excellent photostability.^32–34^ AIEgens have emerged as a novel class of material with practical applications in various areas including OLED, biological imaging and theranostics.^35–40^ Therefore, DTBET systems with AIEgens as dark donors could be a very promising strategy to realize ratiometric sensing by taking advantage of the weak luminescence in solution and the bright emission in the solid state. Amongst the AIE cores, tetraphenylethene (**TPE**) is the most widely used, due to its advantages such as simple synthesis, bright solid-state emission, easy modification to achieve tuneable emission and different functions.

Herein, we report a novel dark through-bond energy transfer strategy based on the connection of a rhodamine moiety with two **TPE** derivatives. Due to the rapid TBET rate, the energy of the dark **TPE** derivatives is completely transferred to the rhodamine moiety before the non-radiative decay and the ETE was as high as 99%. Large pseudo-Stokes shifts of up to 280 nm are achieved. Quantum chemical calculations were conducted to study the DTBET process as well as the structure–property relationship of the DTBET cassettes. Thanks to the emissive features of the **TPE** derivatives in the solid state, ratiometric Hg^2+^ sensors with high selectivity and high sensitivity are developed.

## Results and discussion

### Design and synthesis

**Scheme 1:**
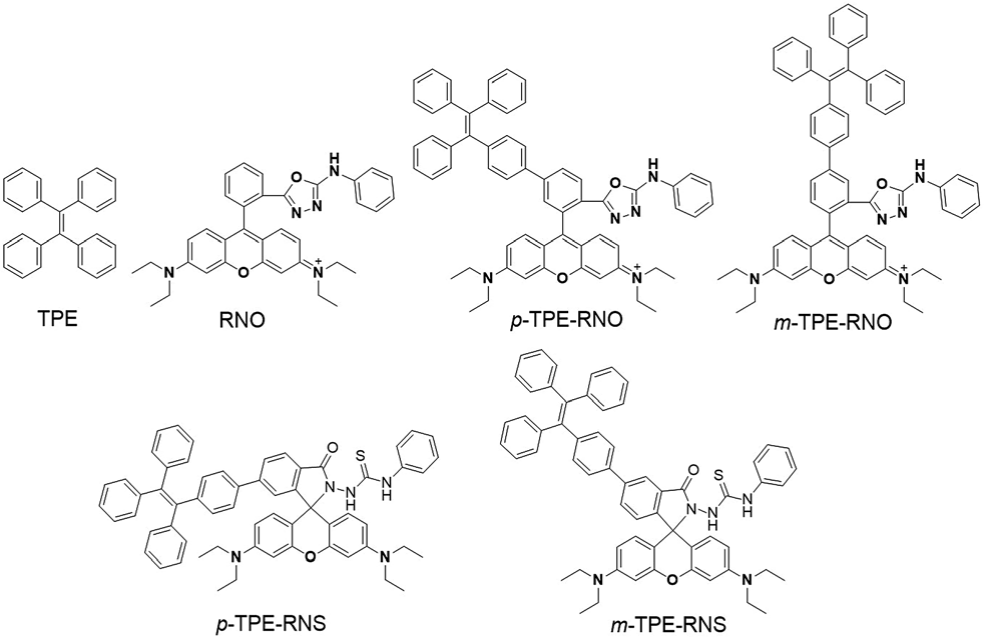
Chemical structures of **TPE**, **RNO**, ***p*/*m*-TPE–RNO** and ***p*/*m*-TPE–RNS**.

The chemical structures and synthetic routes of the DTBET cassette compounds and the acceptor as well as the Hg^2+^ sensors are depicted in Schemes 1 and S1,† respectively. All the compounds are fully characterized by ^1^H NMR and ^13^C NMR spectroscopy and HRMS. **TPE** was chosen as the dark donor because it showed several advantages for the DTBET system: (1) **TPE** is non-emissive in solution due to the fast non-radiative decay, which helps diminish the fluorescence leakage even in the rare case that the TBET efficiency is not high enough;^24^ (2) **TPE** can be easily modified and **TPE** derivatives usually show large Stokes shifts (typically 150–200 nm) and broad emission bands, which are beneficial for constructing DTBET systems with ultra-large pseudo-Stokes shifts and high ETEs; (3) **TPE** derivatives could be highly emissive in the aggregate state due to suppression of the non-radiative decay, which offers an opportunity to develop ratiometric detection through rational design. A rhodamine B derivative was chosen as the acceptor for a couple of reasons: (1) it has large molar absorptivity and a high quantum yield in solution; (2) opening of the spirolactam form of rhodamine has been widely used to develop fluorescent probes for various analytes.^41,42^ The *meso*-phenyl ring exhibits a large twisting angle to the rhodamine core plane due to steric hindrance, which makes the donor and acceptor non-conjugated and the cassettes function as a typical TBET system.^43–45^

**Scheme 2:**
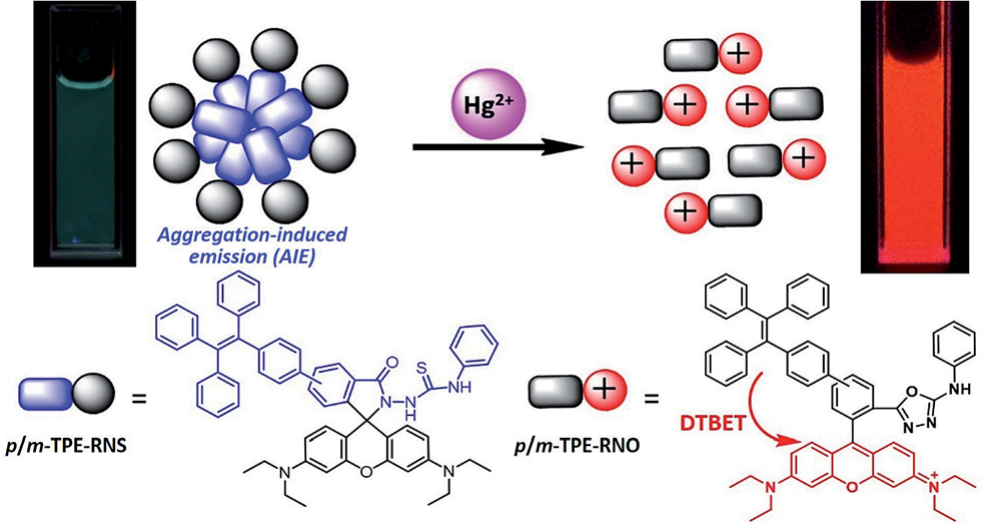
Schematic illustration of the ratiometric Hg^2+^ sensing mechanism of ***p*/*m*-TPE–RNS**.

For application in chemical and biological sensing, a highly specific reaction of thiosemicarbazides to form 1,3,4-oxadiazoles triggered by Hg^2+^ is adopted for recognition of Hg^2+^ (Scheme 2).^46–48^ In the absence of Hg^2+^, the sensors are hydrophobic and tend to form aggregates in water. Due to the non-emissive spirolactam form of rhodamine, only blue emission of the **TPE** aggregate is expected. After treating with Hg^2+^, positively charged rhodamine fluorophores will be generated, whose solubility in water will be greatly improved. As a consequence, the **TPE** emission could not be observed due to the DTBET process and the non-radiative decay while emission of rhodamine will be intensified. Thus, ratiometric Hg^2+^ detection could be realized by this rational design strategy.

### Photophysical properties of the DTBET cassettes

**Fig. 1:**
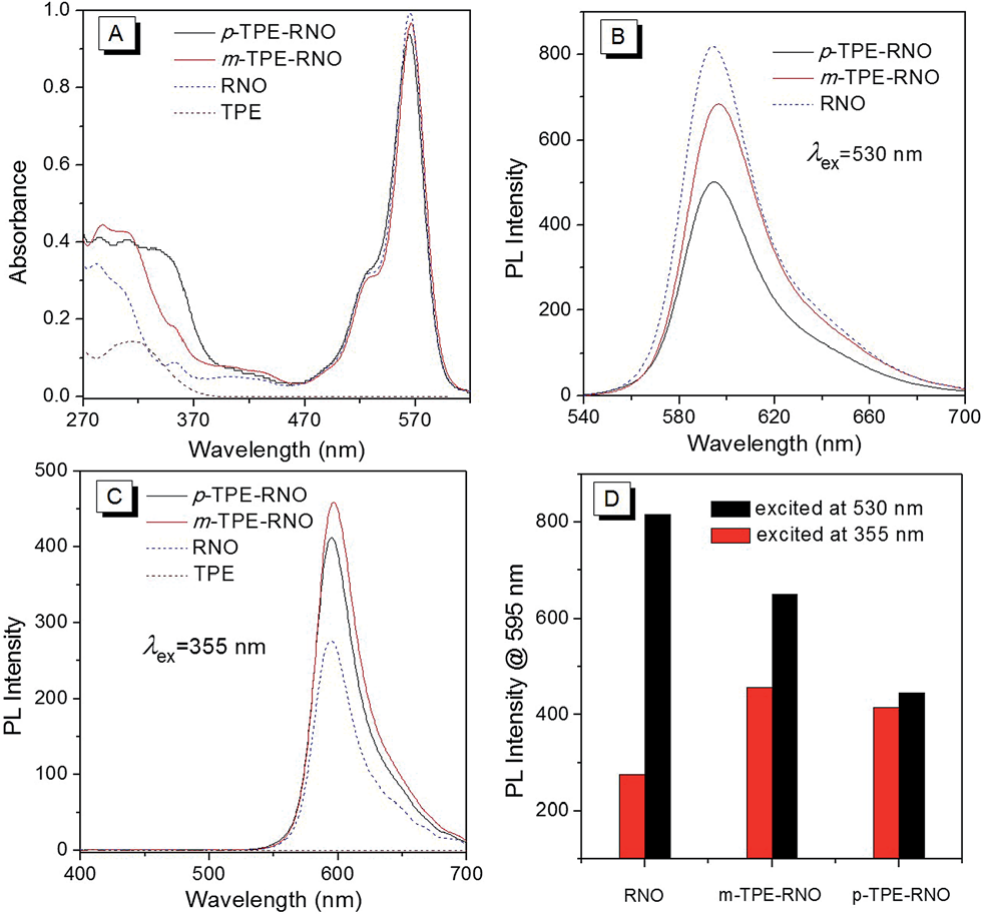
(A) Absorption spectra of **TPE**, **RNO**, ***m*-TPE–RNO** and ***p*-TPE–RNO** in a mixture of CH_3_CN/H_2_O (v/v, 2 : 3). (B) PL spectra of **RNO**, ***m*-TPE–RNO** and ***p*-TPE–RNO** in a mixture of CH_3_CN/H_2_O (v/v, 2 : 3) under excitation at 530 nm. (C) PL spectra of **TPE**, **RNO**, ***m*-TPE–RNO** and ***p*-TPE–RNO** in a mixture of CH_3_CN/H_2_O (v/v, 2 : 3) under excitation at 355 nm. (D) Plot of PL intensity at 595 nm of **RNO**, ***m*-TPE–RNO** and ***p*-TPE–RNO** excited at 355 nm and 530 nm. Dye concentration: 10 µM.

Absorption spectra of the donor (**TPE**), the acceptor (**RNO**) and the DTBET cassettes (***m*-TPE–RNO**/***p*-TPE–RNO**) were recorded in a CH_3_CN/H_2_O mixture (v/v, 2 : 3). As shown in Fig. 1A, the donor **TPE** exhibits an absorption peak at about 315 nm, while the acceptor **RNO** shows an absorption maximum at 565 nm. ***m*-TPE–RNO** shows a major absorption band at around 566 nm and a minor band at about 315 nm, while ***p*-TPE–RNO** exhibits a similar major band centred at 564 nm and a minor band at 355 nm. Both ***m*-TPE–RNO** and ***p*-TPE–RNO** have no significant change in the absorption band of the rhodamine core upon conjugation with **TPE**, which can be attributed to the large twisting angle between the phenyl ring and the rhodamine plane. For the ***m*-TPE–RNO** and ***p*-TPE–RNO**, the **TPE** moiety is at the *meta*- and *para*-position of the oxadiazole group, respectively. ***p*-TPE–RNO** possesses better conjugation than ***m*-TPE–RNO** as suggested by the fact that the minor band is relatively red-shifted from 315 nm in ***m*-TPE–RNO** to 355 nm in ***p*-TPE–RNO**.

Photoluminescence (PL) spectra of each compound in a solution of CH_3_CN/H_2_O (v/v, 2 : 3) were then measured (Fig. 1B–D). When excited at 530 nm, the acceptor **RNO** shows an intense emission peak at around 595 nm with a quantum yield of 21.4%. ***m*-TPE–RNO** and ***p*-TPE–RNO** emit less efficiently with quantum yields of 16.2% and 11.3%, respectively (Fig. 1B). The lower emission intensity in the DTBET cassettes might be attributed to the higher electron density when the **TPE** unit is connected to the *meso*-phenyl ring, which will enhance the photo-induced electron transfer (PET) quenching effect.^49^ However, when the molecules are excited at 355 nm (excitation of the **TPE** moiety), the emission intensity of ***m*-TPE–RNO** and ***p*-TPE–RNO** at 595 nm is obviously larger than that of the **RNO** alone. These results clearly demonstrate that the energy of the **TPE** moiety is successfully transferred to the rhodamine before the fast non-radiative decay. The pseudo-Stokes of ***m*-TPE–RNO** and ***p*-TPE–RNO** are determined to be 280 nm and 240 nm, respectively, which are much larger than the existing DRET systems.^26–28,50^ The energy transfer efficiencies (ETEs) of the DTBET cassettes are calculated to be 99% for ***m*-TPE–RNO** and 69% for ***p*-TPE–RNO**. More importantly, due to the intrinsic fast non-radiative decay of the **TPE** moiety, no **TPE** emission of 480 nm is observed even though the ETE in ***p*-TPE–RNO** is 69%. This result indicates that the DTBET cassettes could be excellent candidates for biological imaging with low background noise.

### Theoretical calculation

**Fig. 2:**
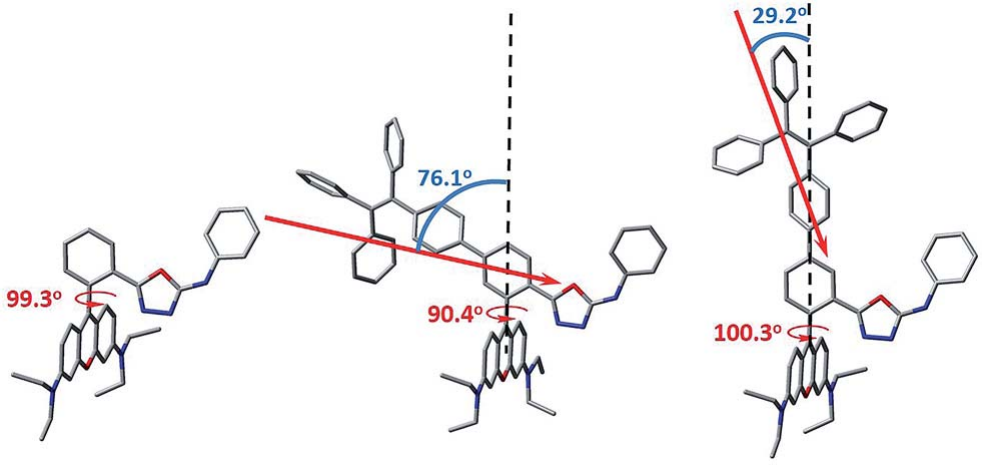
The optimized molecular geometry of **RNO** (left), ***p*-TPE–RNO** (middle) and ***m*-TPE–RNO** (right) and the calculated donor transition dipole moments in the cassettes (red arrows). The linker axes in both DTBET systems are illustrated using black dash lines. Grey: carbon; red: oxygen; blue: nitrogen. Hydrogen atoms are omitted for clarity.

In order to gain more mechanistic insight into the DTBET systems, we performed systematic theoretical calculations using Gaussian 09.^51^ Firstly, the geometries of the acceptor **RNO** and two DTBET cassettes were optimized at the B3LYP/6-31G(d) level. As shown in Fig. 2, the rhodamine core plane and the *meso*-phenyl ring are nearly perpendicular in all molecules with the corresponding dihedral angles of 99.3°, 90.4° and 100.3° for **RNO**, ***p*-TPE–RNO** and ***m*-TPE–RNO**. These results indicate that the π-conjugation between the *meso*-phenyl ring and rhodamine plane is almost blocked. Thus, the donor and acceptor act as two independent fluorophores, which is the characteristic feature for TBET systems. The calculated perpendicular geometries are in good accordance with the experimental data that DTBET cassettes show no change in the absorption of the rhodamine core.

**Fig. 3:**
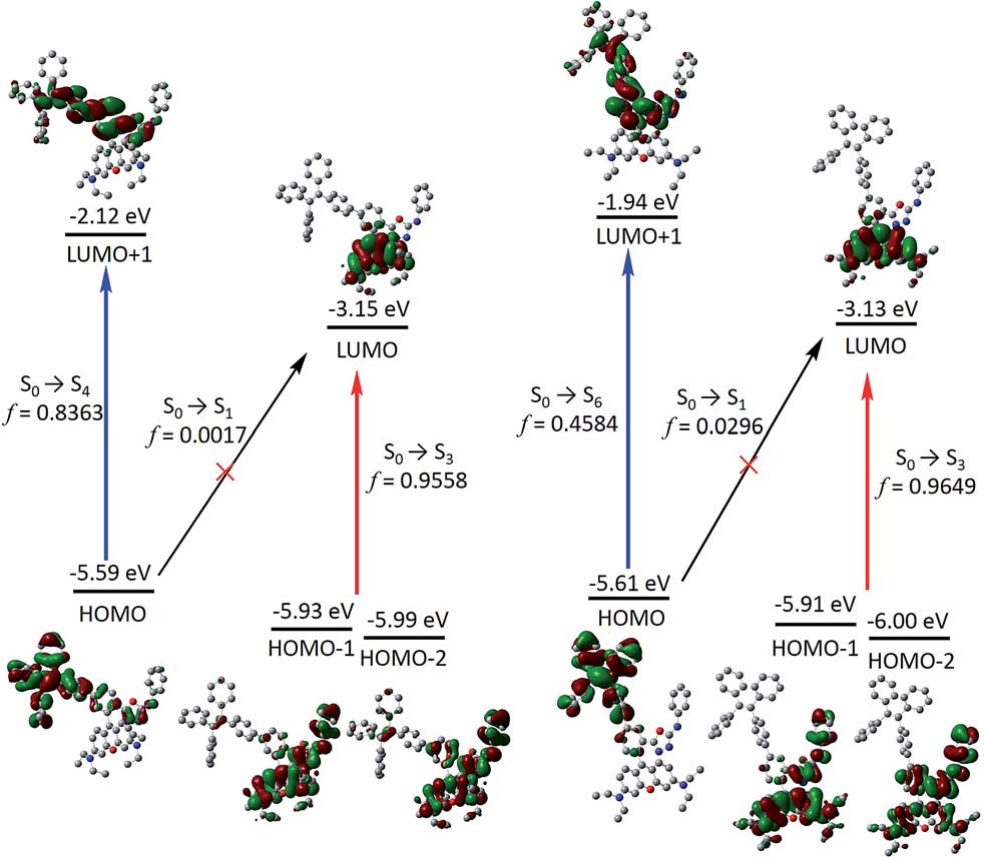
Time dependent B3LYP/6-31G(d) calculated frontier molecular orbitals, energy levels, selected electronic transitions and oscillator strengths of ***p*-TPE–RNO** (left) and ***m*-TPE–RNO** (right). Note that the electron distribution and energy levels of the HOMO–1 and the HOMO–2 in each molecule are very similar. Hydrogen atoms are omitted for clarity.

Next, frontier molecular orbitals (FMOs), energy levels, absorption transitions and oscillator strengths of **RNO**, ***p*-TPE–RNO** and ***m*-TPE–RNO** were calculated and the results are given in Fig. 3, S17 and Table S1,† respectively. To gain a deeper insight into the different ETEs of the two DTBET cassettes, the transition dipole moments of the donor **TPE** parts were calculated as well. From HOMO to LUMO+1 in ***p*-TPE–RNO**, the electron density on the outer three phenyl rings decreased distinctly, while that on the oxadiazole increased greatly (Fig. 3, left). This indicates an intramolecular charge transfer (ICT) process of the donor and the transition orientation is from the outer phenyl rings to the oxadiazole group. Similar results are observed in ***m*-TPE–RNO** with a lower oscillator strength (Fig. 3, right), which is understandable because the electron withdrawing groups are in the *meso*-position of the electron donating group. The orientation of the donor transition moment in ***p*-TPE–RNO** forms an angle of around 76.1° with the linker axis, while that in ***m*-TPE–RNO** exhibits only a tilt angle of 29.2° relative to the linker axis (Fig. 2). It is reported that the energy transfer rate for the transition moment of the donor aligned parallel to the linker axis is faster than the transition moment of the donor aligned perpendicular to the linker axis.^25^ Therefore, the ET rate of ***m*-TPE–RNO** is expected to be faster than that of ***p*-TPE–RNO**, which is in good agreement with the fact that the ETE in ***m*-TPE–RNO** is higher than that in ***p*-TPE–RNO**.

As shown in Fig. 3, two allowed transitions in ***p*-TPE–RNO** are S_0_ → S_3_ (2.613 eV) and S_0_ → S_4_ (3.035 eV). The electron clouds of the HOMO–1, the HOMO–2 and the LUMO are mainly located in the rhodamine part, while those of the HOMO and the LUMO+1 are distributed exclusively on the **TPE** moiety. Therefore, S_0_ → S_3_ and S_0_ → S_4_ can be assigned to the absorption bands of the acceptor rhodamine core and the donor **TPE** moiety, respectively. For compound ***m*-TPE–RNO**, those allowed transitions are S_0_ → S_3_ (2.612 eV) and S_0_ → S_6_ (3.241 eV), which can similarly be assigned to the absorption bands of the rhodamine unit and the **TPE** group. On the other hand, the calculated energy gap of the rhodamine absorption of ***p*-TPE–RNO** (2.613 eV) and ***m*-TPE–RNO** (2.612 eV) are almost the same as that of the acceptor **RNO** (S_0_ → S_2_, 2.615 eV). These theoretical results are in good accordance with the experimental data that ***p*-TPE–RNO** and ***m*-TPE–RNO** show no obvious change in the absorption wavelength maximum of the rhodamine moiety. Collectively, these results confirm that both cassettes are TBET systems.

In the HOMO of ***p*-TPE–RNO**, the electron density is more delocalized to the *meso*-phenyl ring and the oxadiazole group compared to that in the HOMO of ***m*-TPE–RNO**, which further supports that π-conjugation is better and the PET effect is larger in ***p*-TPE–RNO**. The calculated energy gap of the **TPE** absorption in ***p*-TPE–RNO** (S_0_ → S_4_, 3.035 eV) is about 0.21 eV, smaller than that of ***m*-TPE–RNO** (S_0_ → S_6_, 3.241 eV). This is in line with the experimental data in which ***p*-TPE–RNO** shows a longer absorption band at the donor part. In both cassettes, the electron clouds of the HOMOs are predominantly localized on the donor **TPE** moiety, while those of the LUMOs are mainly populated on the rhodamine plane. However, the oscillator strengths (*f*) of S_0_ → S_1_ in ***p*-TPE–RNO** (2.093 eV) and ***m*-TPE–RNO** (2.198 eV) are calculated to be 0.0017 and 0.0296, suggesting that S_0_ → S_1_ transitions are forbidden. This is easy to understand based on the fact that the electron clouds of the HOMOs and the LUMOs in both cassettes have almost no overlapping. Thanks to the TBET process, the energy of the excited **TPE** donor can be transferred to the acceptor and thus the emission from rhodamine is greatly enhanced upon photoexcitation of **TPE**.

### Ratiometric Hg^2+^ sensing

The PL spectra of ***p*-TPE–RNS** and ***m*-TPE–RNS** were collected in CH_3_CN/H_2_O mixtures with different water fractions (Fig. S18†). For ***p*-TPE–RNS**, the PL intensity is quite low when the water fraction is below 50%. Starting from a 55% water fraction, the PL intensity at 485 nm increases along with enhancement of the water fraction and reaches a maximum at 95% water fraction (Fig. S18A and B†). Similarly, the PL spectra of ***m*-TPE–RNS** show weak PL signals at low water fractions. When the water fraction is above 55%, the PL intensity at 480 nm increases but it reaches a maximum at 60% water fraction and then decreases with the increasing water fraction (Fig. S18C and D†). The PL decrement at higher water fraction might be due to the hydrophobicity of ***m*-TPE–RNS**, which forms large aggregates and tends to precipitate out of solution. The particle sizes of ***m*-TPE–RNS** and ***p*-TPE–RNS** at 60% water fraction are determined to be around 159 nm and 182 nm by DLS, respectively (Fig. S19†), and the absolute quantum yields of ***m*-TPE–RNS** and ***p*-TPE–RNS** at 60% water fraction are determined to be 15.2% and 4.1% using an integrating sphere method.

**Fig. 4:**
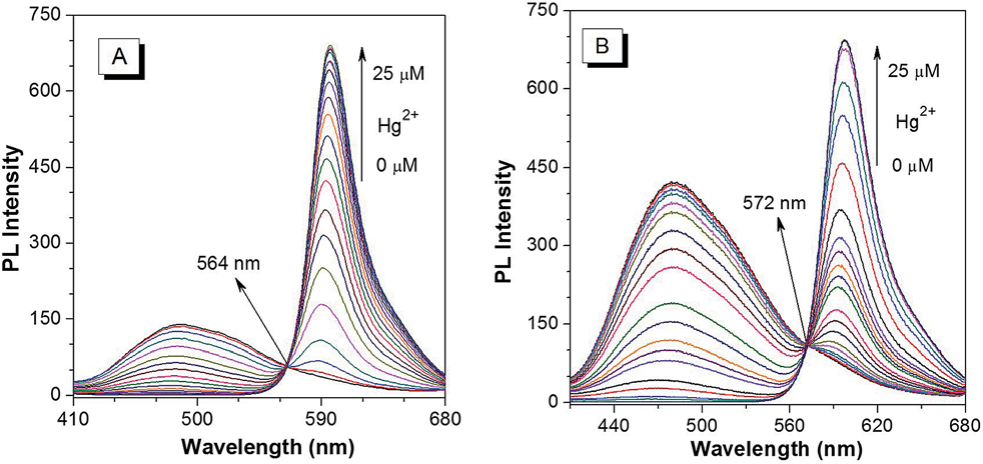
PL spectra of 10 µM (A) ***p*-TPE–RNS** and (B) ***m*-TPE–RNS** in a CH_3_CN/water mixture at 60% water fraction in the presence of different amounts of Hg^2+^. Excitation wavelength: 355 nm.

Next, a PL titration of Hg^2+^ was carried out in the optimized condition of 60% water fraction (Fig. 4 and S20†). As can be seen from Fig. 4A, on increasing the concentration of Hg^2+^, the emission intensity of ***p*-TPE–RNS** at 485 nm dropped and a new peak at 595 nm enhanced gradually. A clear iso-emission point is observed at 564 nm with a large emission change of 110 nm. The PL intensity ratio at 595 nm and 485 nm (*I*_595_/*I*_485_) increased from 0.13 in the absence of Hg^2+^ to 462.9 in the presence of 2 equiv. of Hg^2+^. The ratio enhancement factor is over 3500 fold. The response of ***m*-TPE–RNS** to Hg^2+^ is similar to that of ***p*-TPE–RNS**. A clear iso-emission point at 572 nm is observed. More interestingly, the intensity ratio of *I*_595_/*I*_480_ increased from 0.17 in the absence of Hg^2+^ to 1038.6 in the presence of 2 equiv. of Hg^2+^ with a huge enhancement factor over 6100 fold! Such large ratio enhancements are unprecedented, some of the best existing Hg^2+^ sensors show several-hundred-fold enhancement.^52–54^ The huge ratio enhancement and the well separated emission peaks indicate that the two chemodosimeters could be very sensitive to Hg^2+^.

The detection limits of the two Hg^2+^ sensors are determined to be 1.0 ppb for ***p*-TPE–RNS** and 0.3 ppb for ***m*-TPE–RNS** (3*σ*/slope, Fig. S21†),^55^ which are below the US EPA standard for the maximum Hg^2+^ concentration (2 ppb) allowed in drinking water.^56^ The selectivity is improved distinctly compared to our previous Hg^2+^ sensor based on the AIE mechanism.^57^ The addition of Hg^2+^ will have two effects: (1) it generates the rhodamine core, which leads to the increment of PL intensity at 595 nm and the decrement of PL intensity of the **TPE** moiety due to the fast and efficient TBET process; (2) it reduces the concentration of the DTBET sensor and disassembles the degree of sensor aggregation, which causes further decreasing of the PL intensity of the **TPE** unit. As a result, the DTBET Hg^2+^ sensors show superb ratio increments and exhibit very low detection limits. Combining the AIE and TBET mechanisms, the DTBET mechanism could be a practical design strategy for the development of sensors with high performance.

**Fig. 5:**
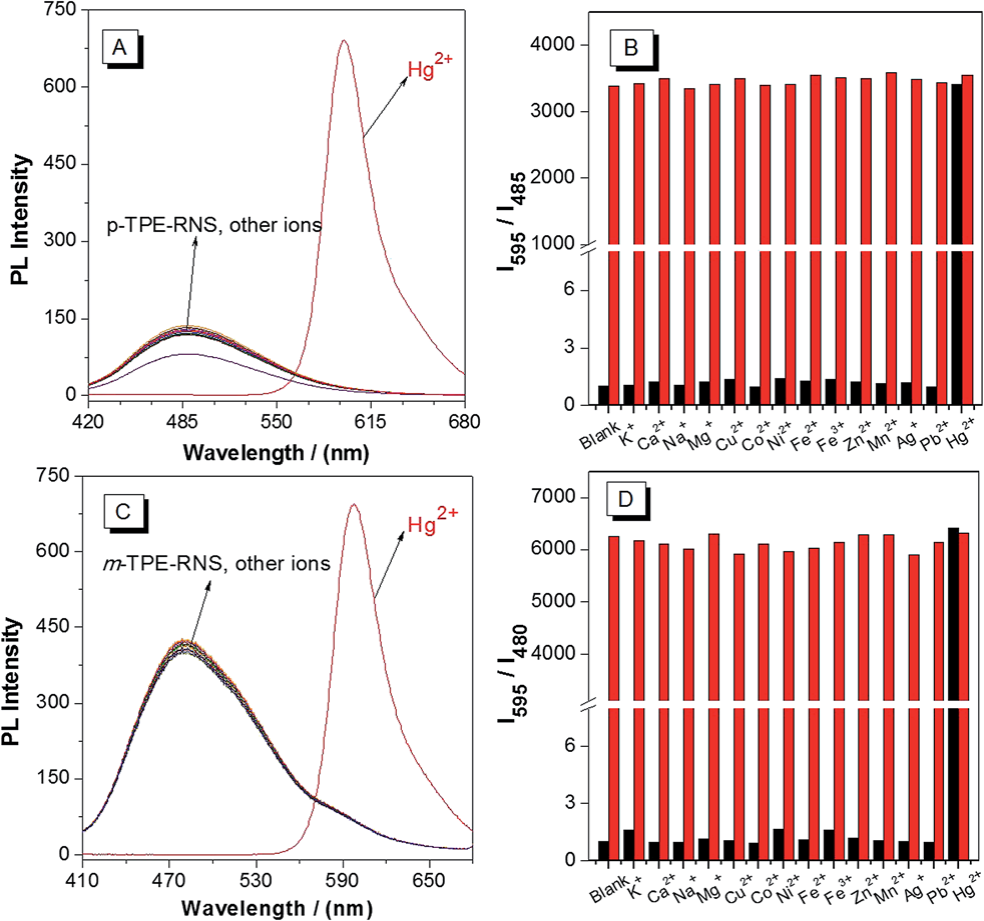
PL spectra of 10 µM ***p*-TPE–RNS** (A) and ***m*-TPE–RNS** (C) in a CH_3_CN/water mixture at 60% water fraction in the presence of different metal ions. K^+^, Ca^2+^, Na^+^, and Mg^2+^ = 1 mM; other metal ions = 20 µM. PL intensity ratio of 10 µM ***p*-TPE–RNS** (B, *I*_595_/*I*_485_) and ***m*-TPE–RNS** (D, *I*_595_/*I*_480_) in a CH_3_CN/water mixture at 60% water fraction. Black bar: blank, or in the presence of different metal ions. Red bar: treated with marked metal ions followed by addition of 20 µM Hg^2+^. The PL intensity ratio of each blank solution is normalized.

PL spectra of the DTBET systems in the presence of different metal ions are collected in a CH_3_CN/H_2_O mixture at 60% water fraction (Fig. 5). For ***p*-TPE–RNS**, the distinct emission change from 485 nm to 595 nm is observed only in the presence of Hg^2+^ (2 equiv.). In contrast, no obvious change in emission is observed upon addition of 2 equiv. of other transition metal ions or 100 equiv. of K^+^, Ca^2+^, Na^+^ and Mg^2+^. Moreover, the ratio of *I*_595_/*I*_485_ induced Hg^2+^ was not affected in the presence of other metal ions. The selectivity of ***m*-TPE–RNS** to Hg^2+^ over other metal ions is similar to that of ***p*-TPE–RNS**. The results indicate that both DTBET systems show a ratiometric Hg^2+^ sensing ability with excellent selectivity, even in the presence of other metal ions.

**Fig. 6:**
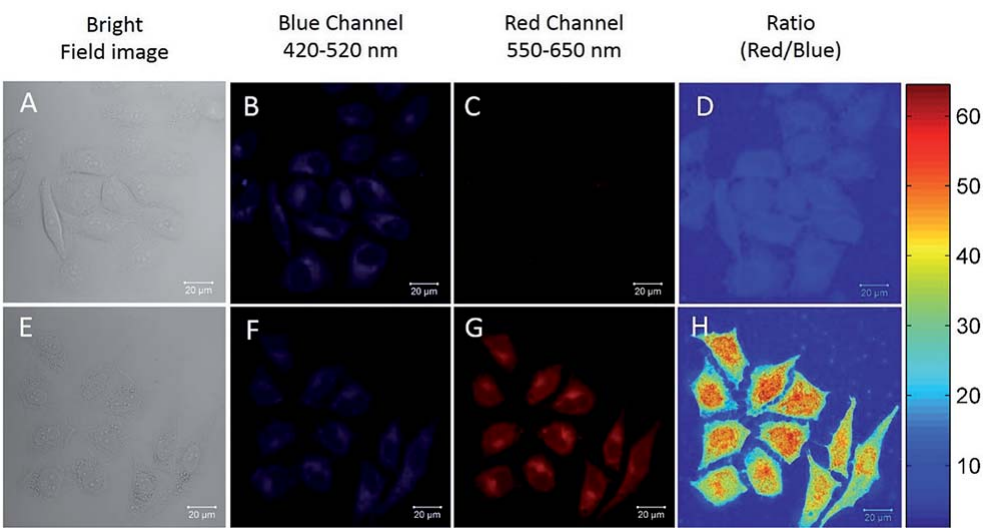
Fluorescent confocal microscopy images of HeLa cells stained with 20 µM of ***p*-TPE–RNS** for 40 min in the absence (A–D) and presence (E–H) of 2 µM Hg^2+^ for 30 min. (A and E) Bright field images; (B and F) blue channel images: 420–520 nm; (C and G) red channel images: 550–650 nm; (D and H) ratio images of red/blue. Excitation wavelength: 405 nm.

The excellent Hg^2+^ sensing performance of the DTBET cassettes in solution encouraged us to evaluate their potential application in biological Hg^2+^ imaging. ***p*-TPE–RNS** is more readily able to penetrate into HeLa cells than ***m*-TPE–RNS** due to its relatively larger polarity and better solubility in water. Therefore, ratiometric Hg^2+^ imaging in HeLa cells is carried out using confocal laser scanning microscopy (Fig. 6). HeLa cells stained with 20 µM of ***p*-TPE–RNS** for 40 min in the absence of Hg^2+^ show moderate emission intensity in the blue channel (420–520 nm) and weak fluorescent signals in the red channel (550–650 nm), indicating a very low level of intracellular Hg^2+^ (Fig. 6A–D). After washing followed by incubation with 2 µM Hg^2+^ for 30 min, a decrement in the blue channel and a distinct enhancement in the red channel are detected. The green to orange colour of the merged image indicated a distinct increment in the intracellular Hg^2+^ level after Hg^2+^ incubation (Fig. 6E–H). A MTT assay was also carried out and no obvious cytotoxicity to HeLa cells is observed with the ***p*-TPE–RNS** concentration up to 100 µM (Fig. S22†). The results confirmed that ***p*-TPE–RNS** could be a good ratiometric imaging agent for Hg^2+^ detection in living cells.

## Conclusions

A novel DTBET mechanism was proposed and two DTBET cassettes were designed and synthesized, carrying AIE-active **TPE** derivatives as the dark donors and a rhodamine core as the acceptor. The DTBET mechanism was illustrated by studying the photophysical properties and by theoretical calculations. The DTBET cassettes showed pseudo-Stokes shifts of up to 280 nm, which is larger than the existing DRET system due to the lower requirement for substantial spectral overlapping of the DTBET mechanism. The donor energy was efficiently transferred to the acceptor due to the fast TBET process, making the DTBET system a good candidate for biological imaging with low background interference. In addition, ratiometric Hg^2+^ detection was realized. The novel DTBET Hg^2+^ sensors showed well separated emission peaks with large emission wavelength changes up to 115 nm, making them highly sensitive with detection limits as low as 0.3 ppb. Their potential as practical biological imaging agents was further confirmed by confocal fluorescence microscopy in HeLa cells. This study provides a new design strategy for the development of sensors with high performance, which could be a powerful tool for the tracking and exploration of various biological and physiological processes. Further development of the DTBET cassettes library with tunable excitation/emission and DTBET-based sensors for other biological species is ongoing in our group.

## Experimental

### Materials and general methods

All the solvents used in preparation and purification were of analytic grade. THF was distilled under normal pressure under nitrogen immediately prior to use. All the solvents used in the spectroscopic study were of HPLC grade and Milli-Q water was used as deionized water. All the chemicals were purchased from J&K Chemicals or Sigma-Aldrich and were used as received without further purification. The stock solutions of metal ions were prepared from KCl, CaCl_2_, NaCl, MgCl_2_·6H_2_O, CuSO_4_, MnCl_2_, CoCl_2_·6H_2_O, Zn(NO_3_)_2_·7H_2_O, NiCl_2_·6H_2_O, FeCl_2_, FeCl_3_, CdCl_2_·2.5H_2_O, AgNO_3_, Pb(NO_3_)_2_ and HgCl_2_ with doubly distilled water. ^1^H and ^13^C NMR spectra were measured on a Bruker ARX 400 NMR spectrometer using CDCl_3_ or CD_2_Cl_2_ as the solvent and tetramethylsilane (TMS) as an internal reference. UV absorption spectra were taken on a Milton Roy Spectronic 3000 array spectrophotometer. Photoluminescence (PL) spectra were recorded on a Perkin-Elmer LS 55 spectrofluorometer. Solid state quantum efficiency was measured using a Hamamatsu C11347 Quantaurus-QY integrating sphere at an excitation wavelength of 530 nm. High-resolution mass spectra (HRMS) were obtained on a GCT Premier CAB 048 mass spectrometer operated in MALDI-TOF mode. Particle sizes of the nano-aggregates were determined using a ZETA-Plus potential analyzer.

### Syntheses and characterization

Synthetic routes of all the compounds, detailed synthetic procedures and characterization of the intermediate compounds are shown in the ESI.†

#### Synthesis of ***p*-TPE–RNS**

Isothiocyanatobenzene (135 mg, 1.0 mmol), ***p*-TPE–RHZ** (197 mg, 0.25 mmol), and TEA (0.1 mL) were dissolved in 10 mL DMF, the reaction mixture was stirred at room temperature under N_2_ protection for 8 h. The solvent was removed under vacuum, and the resulting residue was purified by column chromatography on silica gel (hexane/CH_2_Cl_2_/ethyl acetate, 2 : 1 : 1 v/v/v) to give ***p*-TPE–RNS** (210 mg, 91%). ^1^H NMR (400 MHz, CDCl_3_): *δ* 8.02 (d, *J* = 8.0 Hz, 1H), 7.77 (dd, *J* = 1.6 Hz, *J* = 8.0 Hz, 1H), 7.53 (s, 1H), 7.42 (d, *J* = 1.6 Hz, 1H), 7.30 (d, *J* = 8.0 Hz, 2H), 7.19 (t, *J* = 3.6 Hz, 2H), 7.10–6.97 (m, 21H), 6.53 (d, *J* = 8.8 Hz, 2H), 6.45 (d, *J* = 2.8 Hz, 2H), 6.30 (dd, *J* = 2.8 Hz, *J* = 8.8 Hz, 2H), 3.35 (q, *J* = 6.8 Hz, 8H), 1.17 (t, *J* = 6.8 Hz, 12H); ^13^C NMR (100 MHz, CDCl_3_): *δ* 182.8, 167.1, 154.3, 150.9, 149.4, 147.0, 144.2, 143.5, 143.4, 141.6, 140.1, 137.7, 137.3, 132.0, 131.4, 131.3, 128.3, 127.9, 127.7, 127.6, 126.6, 126.5, 126.1, 125.2, 124.2, 122.8, 108.4, 104.2, 98.3, 67.3, 44.4, 12.6. HRMS: calc. for [M^+^] 921.4076, found 921.4098.

#### Synthesis of ***m*-TPE–RNS**

The synthetic procedure was similar to that of ***p*-TPE–RNS** but used ***m*-TPE–RHZ** as the starting material. Yield: 92%. ^1^H NMR (400 MHz, CDCl_3_): *δ* 8.20 (s, 1H), 7.85 (d, *J* = 8.0 Hz, 1H), 7.53 (s, 1H), 7.45 (d, *J* = 8.0 Hz, 2H), 7.31 (d, *J* = 8.0 Hz, 2H), 7.21–7.06 (m, 22H), 6.98 (s, 1H), 6.54 (d, *J* = 8.8 Hz, 2H), 6.47 (d, *J* = 2.0 Hz, 2H), 6.31 (dd, *J* = 2.0 Hz, *J* = 8.8 Hz, 2H), 3.36 (q, *J* = 7.2 Hz, 8H), 1.19 (t, *J* = 7.2 Hz, 12H); ^13^C NMR (100 MHz, CDCl_3_): *δ* 182.7, 167.2, 154.3, 149.4, 148.8, 143.8, 143.6, 143.5, 142.0, 141.6, 140.2, 137.7, 137.3, 133.0, 132.1, 131.4, 131.3, 131.2, 129.7, 129.6, 128.3, 127.9, 127.8, 127.7, 127.6, 126.7, 126.6, 126.4, 126.1, 125.8, 125.1, 125.0, 124.2, 121.9, 108.4, 104.2, 98.4, 67.2, 44.4, 12.6. HRMS: calc. for [M^+^] 921.4076, found 921.4036.

#### Synthesis of ***p*-TPE–RNO**

***p*-TPE–RNS** (92 mg, 0.1 mmol) was dissolved in 5 mL CH_3_CN, HgCl_2_ (54 mg, 0.2 mmol) was added and the mixture was left stirring for 6 h at room temperature. After removing the solvent, the residue was purified by column chromatography on silica gel (DCM/MeOH, 20 : 1, v/v) to give ***p*-TPE–RNO** (80 mg, 90%). ^1^H NMR (400 MHz, CD_2_Cl_2_): *δ* 11.20 (br, 1H), 8.30 (s, *J* = 8.0 Hz, 1H), 7.88 (d, *J* = 8.4 Hz, 1H), 7.73 (d, *J* = 8.4 Hz, 2H), 7.48 (s, 1H), 7.42 (d, *J* = 8.0 Hz, 2H), 7.21–7.02 (m, 21H), 6.89 (t, *J* = 7.6 Hz, 2H), 6.78–6.76 (m, 4H), 3.56 (q, *J* = 7.2 Hz, 8H), 1.28 (t, *J* = 7.2 Hz, 12H); ^13^C NMR (100 MHz, CDCl_3_): *δ* 160.5, 158.1, 157.7, 156.0, 155.6, 144.4, 143.6, 143.5, 143.4, 142.1, 141.8, 140.1, 139.0, 136.3, 132.0, 131.4, 131.2, 131.1, 131.0, 130.5, 129.2, 128.6, 128.0, 127.8, 127.7, 127.6, 126.6, 126.2, 122.2, 121.7, 117.7, 114.1, 113.9, 96.3, 46.0, 12.3. HRMS: calc. for [M^+^] 888.4272, found 888.4253.

#### Synthesis of ***m*-TPE–RNO**

The synthetic procedure was similar to that of ***p*-TPE–RNO** but used ***m*-TPE–RNS** as the starting material. Yield: 95%. ^1^H NMR (400 MHz, CDCl_3_): *δ* 10.99 (br, 1H), 8.49 (s, 1H), 7.79 (d, *J* = 8.0 Hz, 1H), 7.73 (d, *J* = 8.0 Hz, 2H), 7.54 (d, *J* = 8.0 Hz, 2H), 7.27 (d, *J* = 8.0 Hz, 1H), 7.20–7.06 (m, 21H), 6.86 (t, *J* = 7.2 Hz, 1H), 6.79 (d, *J* = 9.6 Hz, 2H), 6.72 (s, 2H), 3.52 (q, *J* = 6.8 Hz, 8H), 1.28 (t, *J* = 6.8 Hz, 12H); ^13^C NMR (100 MHz, CDCl_3_): *δ* 160.7, 157.9, 157.4, 156.1, 155.4, 144.4, 143.6, 143.4, 143.0, 141.6, 140.3, 138.8, 136.2, 132.2, 131.5, 131.4, 131.3, 131.2, 130.9, 128.6, 128.5, 127.9, 127.8, 127.7, 126.8, 126.7, 126.6, 126.5, 124.2, 121.7, 118.0, 114.2, 113.8, 96.5, 46.1, 12.7. HRMS: calc. for [M^+^] 888.4272, found 888.4244.

### Calculation of DTBET efficiency

In a typical FRET system, the ETE can usually be calculated using the following equation:

1 *E* = 1 – (*I*_DA_/*I*_D_)

in which *I*_DA_ is the integral of the emission spectra of the donor in the presence of an acceptor, while *I*_D_ is that of the donor in the absence of an acceptor. However, in the dark energy transfer system, both *I*_DA_ and *I*_D_ could not be measured. Therefore, we proposed an alternative method to calculate the ETEs for the dark energy transfer systems.^1^

First, we consider the situation of no energy transfer from the donor to the acceptor, which means that the energy transfer efficiency *E* = 0% and only the emission induced by the acceptor itself will be observed. Due to the different quantum yields of the acceptor and the cassette and based on the fact that no absorption of the donor occurs at the absorption peak of the acceptor, the integral of the emission spectra (*I*_0%_) of the cassette excited at the donor absorption peak can be calculated from the following equation:

2 *I*_0%_ = (*Φ*_C_/*Φ*_A_) × *I*_A_

in which *Φ*_A_ is the quantum yield of the acceptor excited at the absorption peak of the acceptor, *Φ*_C_ is the quantum yield of the cassette excited at the same wavelength of *Φ*_A_, and *I*_A_ is the integral of the emission spectra of the acceptor excited at the donor absorption peak.

Next, we assume that the energy absorbed by the donor was completely transferred to the donor, which means that the energy transfer efficiency *E* = 100%. As a result, the observed emission spectrum should be the sum of the emission induced by the absorption of the acceptor and the emission originating from the energy transfer after the absorption from the donor. The integral of the emission spectra (*I*_100%_) of the cassette can be calculated using the following equation:

3 *I*_100%_ = (*Φ*_C_/*Φ*_A_) × *I*_A_ + (*A*_C_ – *A*_A_)/*A*_A_ × (*Φ*_C_/*Φ*_A_) × *I*_A_ = (*A*_C_/*A*_A_) × (*Φ*_C_/*Φ*_A_) × *I*_A_

where *A*_C_ is the absorbance of the cassette at the wavelength of the donor absorption peak, and *A*_A_ is the absorbance of the acceptor at the same wavelength of *A*_C_.

Finally, ruling out the emission originating from the acceptor, the real increment of the emission and the theoretical emission increment can be calculated as *I* – *I*_0%_ and *I*_100%_ – *I*_0%_, respectively. Therefore, the energy transfer efficiency (*E*) of the cassette can be calculated as:

4 *E* = (*I* – *I*_0%_)/(*I*_100%_ – *I*_0%_)

in which *I* is the measured integral of the emission spectra of the cassette excited at the same wavelength of *I*_0%_.

### Theoretical calculation

All the quantum chemical calculations were carried out using Gaussian 09. Geometry optimizations were performed using density functional theory (DFT) at the B3LYP level of theory employing the 6-31G(d) basis set. Solvent effects (THF) were accounted for using the PCM solvation model. All the torsion angles were measured using Gaussview 5.0.8. The UV-vis absorption of all the compounds was calculated using the time-dependant density functional theory (TDDFT) based on the optimized ground state geometry (S_0_ state). All the figures of the electronic distribution of the frontier molecular orbitals (FMOs) were drawn using Gaussview 5.0.8.

### Confocal fluorescence imaging

HeLa cells were cultured in MEM (minimum essential medium) supplemented with 10% FBS (fetal bovine serum) in an atmosphere of 5% CO_2_ at 37 °C. Ratiometric imaging of the HeLa cells was performed using a laser scanning confocal fluorescence microscope (Zeiss LSM7 DUO). For the ratiometric fluorescence imaging, the blue channel = 420–520 nm, the red channel = 550–650 nm and the excitation wavelength was 405 nm. Before imaging, the cells were washed with phosphate buffered saline (PBS) (pH 7.4) solution three times. Ratiometric images were processed using the imaging analysis program MATLAB. Data for each pixel were calculated from *I*_red_/*I*_blue_ and the reconstructed pseudo colours in the picture represent the different ratios (*I*_red_/*I*_blue_) in the exact position.
