# Supplementary material for: AIEgens for dark through-bond energy transfer: design, synthesis, theoretical study and application in ratiometric Hg2+ sensing
Source: Chem Sci. 2016 Nov 15;8(3):2047–55. doi: 10.1039/c6sc04206f (PMC5398306; doi:10.1039/c6sc04206f)
Supplement: Supplementary file 3 [file SC-008-C6SC04206F-s003.pdf]

Electronic Supplementary Material (ESI) for Chemical Science.
This journal is © The Royal Society of Chemistry 2016

Supporting Information
for

AIEgens for dark through-bond energy transfer: design, synthesis, theoretical study and its application in ratiometric Hg^{2+} sensing

*Yuncong Chen,‡ Weijie Zhang,‡ Yuanjing Cai, Ryan T. K. Kwok, Yubing Hu, Jacky W. Y. Lam, Xinggui Gu, Zikai He, Zheng Zhao, Xiaoyan Zheng, Bin Chen, Chen Gui and Ben Zhong Tang**

1. Synthetic Routes and Characterizations.

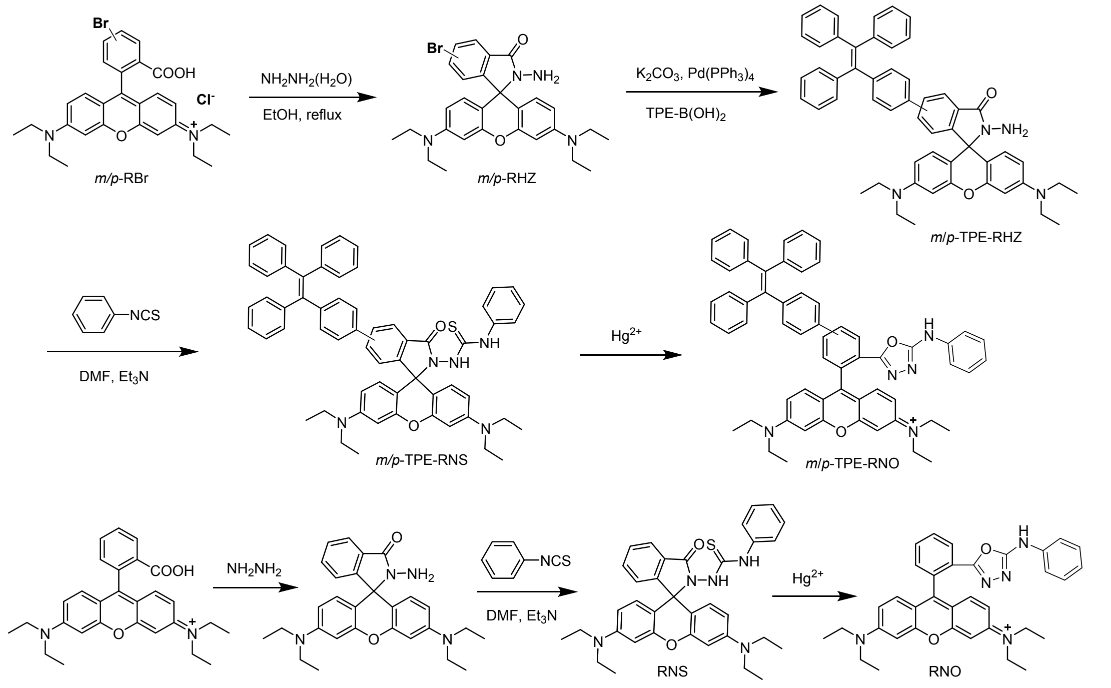

Scheme S1. Synthetic route of *m*/*p*-TPE-RNS, *m*/*p*-TPE-RNO and control compounds RNS, RNO.

Synthesis of *p*-RHZ Compound *p*-RBr were prepared according the reported procedure.[i] Compound *p*-RBr (0.535 g, 1.0 mmol) was dissolved in 20 mL ethanol and 2 mL Hydrazine monohydrate was added. The reaction mixture was refluxed with stirring for 6 h and then evaporated in vacuo. The residue was purified by column chromatography on silica gel (Hexane/ ethyl acetate, 5:1 to 3:1, v/v) to give *p*-RHZ (0.35 g) as white powder, yield, 64%. ^{1}H NMR (400 MHz, $CDCl_3$): δ 7.82 (d, *J* = 8.0 Hz, 1H), 7.60 (dd, *J* = 1.6 Hz, *J* = 8.0 Hz, 1H), 7.26 (d, *J* = 1.6 Hz, 1H), 6.48 (d, *J* = 8.8 Hz, 2H), 6.44 (d, *J* = 1.6 Hz, 2H), 6.32 (dd, *J* = 1.6 Hz, *J* = 8.8 Hz, 2H), 3.62 (s, 2H), 3.37 (q, *J* = 7.2 Hz, 8H), 1.20 (t, *J* = 7.2 Hz, 12H); ^{13}C NMR (100 MHz, $CDCl_3$): δ 165.3, 153.8, 153.3, 149.1, 131.7, 128.8, 128.0, 127.2, 124.6, 108.1, 103.6, 98.0, 65.8, 44.4, 12.6; HRMS: Calc. for [M+H$^+$] 535.1703, found 535.1729.

Synthesis of m-RHZ Synthetic procedure is similar to that of *p*-RHZ using *m*-RBr as the starting material, yield, 68%. ^{1}H NMR (400 MHz, $CDCl_3$): δ 8.09 (d, *J* = 2.0 Hz, 1H), 7.59 (dd, *J* = 1.0 Hz, *J* = 7.6 Hz, 1H), 7.01 (d, *J* = 7.6 Hz, 1H), 6.47 (d, *J* = 8.4 Hz, 2H), 6.43 (d, *J* = 2.4 Hz, 2H), 6.32 (dd, *J* = 2.4 Hz, *J* = 8.4 Hz, 2H), 3.63 (s, 2H), 3.36 (q, *J* = 7.2 Hz, 8H), 1.19 (t, *J* = 7.2 Hz, 12H); ^{13}C NMR (100 MHz, $CDCl_3$): δ 164.6, 153.8, 150.2, 149.0, 135.5, 132.0, 128.0, 126.1, 125.6, 122.2, 108.1, 103.7, 98.0, 66.0, 44.4, 12.6; HRMS: Calc. for [M] 534.1630, found 534.1635.

Synthesis of *p*-TPE-RHZ Compounds *p*-RHZ (268 mg, 0.5 mmol), TPE-B(OH)$_2$ (188 mg, 0.5 mmol), $Pd(PPh_3)_4$ (20 mg, 0.017 mmol) and K_2CO_3 (138 mg, 1.0 mmol) were placed in a 100 mL two neck flask. After vacuum and filling N_2 for 3 times, 25 mL of THF and 10 mL H_2O were added.

The reaction mixture was left refluxing overnight. The THF solvent was removed under vacuum, distract with DCM and H_2O. After removing DCM, the resulting residue was purified by column chromatography on silica gel (Hexane/CH_2Cl_2/ethyl acetate, 2:1:1 v/v/v) to give *p*-TPE-RHZ (324 mg, 82%). ^1H NMR (400 MHz, $CDCl_3$): δ 7.95 (d, *J* = 8.0 Hz, 1H), 7.64 (dd, *J* = 1.6 Hz, *J* = 8.0 Hz, 1H), 7.25 (d, *J* = 1.6 Hz, 1H), 6.98-7.07 (m, 19H), 6.49 (d, *J* = 8.8 Hz, 2H), 6.42 (d, *J* = 2.4 Hz, 2H), 6.30 (dd, *J* = 2.4 Hz, *J* = 8.8 Hz, 2H), 3.61 (s, 2H), 3.35 (q, *J* = 6.8 Hz, 8H), 1.17 (t, *J* = 6.8 Hz, 12H); ^{13}C NMR (100 MHz, $CDCl_3$): δ 166.0, 153.8, 152.2, 148.9, 145.1, 143.6, 141.3, 140.2, 137.8, 131.8, 131.4, 131.3, 131.2, 128.9, 128.2, 127.8, 127.7, 127.6, 127.0, 126.6, 126.5, 123.3, 122.1, 108.1, 104.5, 97.9, 66.1, 44.4, 12.6; HRMS: Calc. for [M] 786.3934, found 786.3986.

Synthesis of *m*-TPE-RHZ Synthetic procedure is similar to that of *p*-TPE-RHZ using *m*-RHZ as the starting material, yield, 85%. ^1H NMR (400 MHz, $CDCl_3$): δ 8.12 (d, *J* = 2.4 Hz, 1H), 7.65 (dd, *J* = 2.4 Hz, *J* = 8.0 Hz, 1H), 7.39 (d, *J* = 8.0 Hz, 2H), 7.04-7.11 (m, 19H), 6.50 (d, *J* = 8.8 Hz, 2H), 6.43 (d, *J* = 2.4 Hz, 2H), 6.30 (dd, *J* = 2.4 Hz, *J* = 8.8 Hz, 2H), 3.63 (s, 2H), 3.35 (q, *J* = 6.8 Hz, 8H), 1.17 (t, *J* = 6.8 Hz, 12H); ^{13}C NMR (100 MHz, $CDCl_3$): δ 166.1, 153.9, 150.3, 148.9, 143.7, 143.6, 143.5, 143.3, 141.4, 141.0, 140.4, 137.9, 131.9, 131.4, 131.3, 131.2, 130.6, 128.2, 127.8, 127.7, 127.6, 127.0, 126.6, 126.5, 126.4, 126.3, 124.1, 121.2, 108.1, 104.5, 98.0, 65.9, 44.4, 12.6; HRMS: Calc. for [M] 786.3934, found 786.3918.

Synthesis of *p*-TPE-RNS Isothiocyanatobenzene (135 mg, 1.0 mmol), *p*-TPE-RHZ (197 mg, 0.25 mmol), and TEA (0.1 mL) were dissolved in 10 mL DMF, the reaction mixture was stirred at room temperature under N_2 protection for 8 hours. The solvent were removed under vacuum, and the resulting residue was purified by column chromatography on silica gel (Hexane/CH_2Cl_2/ethyl acetate, 2:1:1 v/v/v) to give *p*-TPE-RNS (210 mg, 91%). ^1H NMR (400 MHz, $CDCl_3$): δ 8.02 (d, *J* = 8.0 Hz, 1H), 7.77 (dd, *J* = 1.6 Hz, *J* = 8.0 Hz, 1H), 7.53 (s, 1H), 7.42 (d, *J* = 1.6 Hz, 1H), 7.30 (d, *J* = 8.0 Hz, 2H), 7.19 (t, *J* = 3.6 Hz, 2H), 6.97-7.10 (m, 21 H), 6.53 (d, *J* = 8.8 Hz, 2H), 6.45 (d, *J* = 2.8 Hz, 2H), 6.30 (dd, *J* = 2.8 Hz, *J* = 8.8 Hz, 2H), 3.35 (q, *J* = 6.8 Hz, 8H), 1.17 (t, *J* = 6.8 Hz, 12H); ^{13}C NMR (100 MHz, $CDCl_3$): δ 182.8, 167.1, 154.3, 150.9, 149.4, 147.0, 144.2, 143.5, 143.4, 141.6, 140.1, 137.7, 137.3, 132.0, 131.4, 131.3, 128.3, 127.9, 127.7, 127.6, 126.6, 126.5, 126.1, 125.2, 124.2, 122.8, 108.4, 104.2, 98.3, 67.3, 44.4, 12.6; HRMS: Calc. for [M] 921. 4076, found 921. 4098.

Synthesis of *m*-TPE-RNS Synthetic procedure is similar to that of *p*-TPE-RNS using *m*-TPE-RHZ as the starting material, yield, 92%. ^1H NMR (400 MHz, $CDCl_3$): δ 8.20 (s, 1H), 7.85 (d, *J* = 8.0 Hz, 1H), 7.53 (s, 1H), 7.45 (d, *J* = 8.0 Hz, 2H), 7.31 (d, *J* = 8.0 Hz, 2H), 7.06-7.21 (m, 22 H), 6.98 (s, 1H), 6.54 (d, *J* = 8.8 Hz, 2H), 6.47 (d, *J* = 2.0 Hz, 2H), 6.31 (dd, *J* = 2.0 Hz, *J* = 8.8 Hz, 2H), 3.36 (q, *J* = 7.2 Hz, 8H), 1.19 (t, *J* = 7.2 Hz, 12H); ^{13}C NMR (100 MHz, $CDCl_3$): δ 182.7, 167.2, 154.3, 149.4, 148.8, 143.8, 143.6, 143.5, 142.0, 141.6, 140.2, 137.7, 137.3, 133.0, 132.1, 131.4, 131.3, 131.2, 129.7, 129.6, 128.3, 127.9, 127.8, 127.7, 127.6, 126.7, 126.6, 126.4, 126.1, 125.8, 125.1, 125.0, 124.2, 121.9, 108.4, 104.2, 98.4, 67.2, 44.4, 12.6; HRMS: Calc. for [M] 921. 4076, found 921. 4036.

Synthesis of *p*-TPE-RNO Compound *p*-TPE-RNS (92 mg, 0.1 mmol) was dissolved in 5 mL CH_3CN, $HgCl_2$ (54 mg, 0.2 mmol) was added and the mixture was left stirring for 6h at room

temperature. After removing the solvent, the residue was purified by column chromatography on silica gel (DCM/MeOH, 20:1, v/v) to give *p*-TPE-RNO (80 mg, 90%). ^{1}H NMR (400 MHz, CD_2Cl_2): δ 11.20 (br, 1H), 8.30 (s, *J* = 8.0 Hz, 1H), 7.88 (d, *J* = 8.4 Hz, 1H), 7.73 (d, *J* = 8.4 Hz, 2H), 7.48 (s, 1H), 7.42 (d, *J* = 8.0 Hz, 2H), 7.02-7.21 (m, 21 H), 6.89 (t, *J* = 7.6 Hz, 2H), 6.76 - 6.78 (m, 4H), 3.56 (q, *J* = 7.2 Hz, 8H), 1.28 (t, *J* = 7.2 Hz, 12H); ^{13}C NMR (100 MHz, $CDCl_3$): δ 160.5, 158.1, 157.7, 156.0, 155.6, 144.4, 143.6, 143.5, 143.4, 142.1, 141.8, 140.1, 139.0, 136.3, 132.0, 131.4, 131.2, 131.1, 131.0, 130.5, 129.2, 128.6, 128.0, 127.8, 127.7, 127.6, 126.6, 126.2, 122.2, 121.7, 117.7, 114.1, 113.9, 96.3, 46.0, 12.3; HRMS: Calc. for [M] 888. 4272, found 888. 4253.

Synthesis of *m*-TPE-RNO Synthetic procedure is similar to that of *p*-TPE-RNO using *m*-TPE-RNS as the starting material, yield, 95%. ^{1}H NMR (400 MHz, $CDCl_3$): δ 10.99 (br, 1H), 8.49 (s, 1H), 7.79 (d, *J* = 8.0 Hz, 1H), 7.73 (d, *J* = 8.0 Hz, 2H), 7.54 (d, *J* = 8.0 Hz, 2H), 7.27 (d, *J* = 8.0 Hz, 1H), 7.06-7.20 (m, 21 H), 6.86 (t, *J* = 7.2 Hz, 1H), 6.79 (d, *J* = 9.6 Hz, 2H), 6.72 (s, 2H), 3.52 (q, *J* = 6.8 Hz, 8H), 1.28 (t, *J* = 6.8 Hz, 12H); ^{13}C NMR (100 MHz, $CDCl_3$): δ 160.7, 157.9, 157.4, 156.1, 155.4, 144.4, 143.6, 143.4, 143.0, 141.6, 140.3, 138.8, 136.2, 132.2, 131.5, 131.4, 131.3, 131.2, 130.9, 128.6, 128.5, 127.9, 127.8, 127.7, 126.8, 126.7, 126.6, 126.5, 124.2, 121.7, 118.0, 114.2, 113.8, 96.5, 46.1, 12.7; HRMS: Calc. for [M] 888. 4272, found 888. 4244.

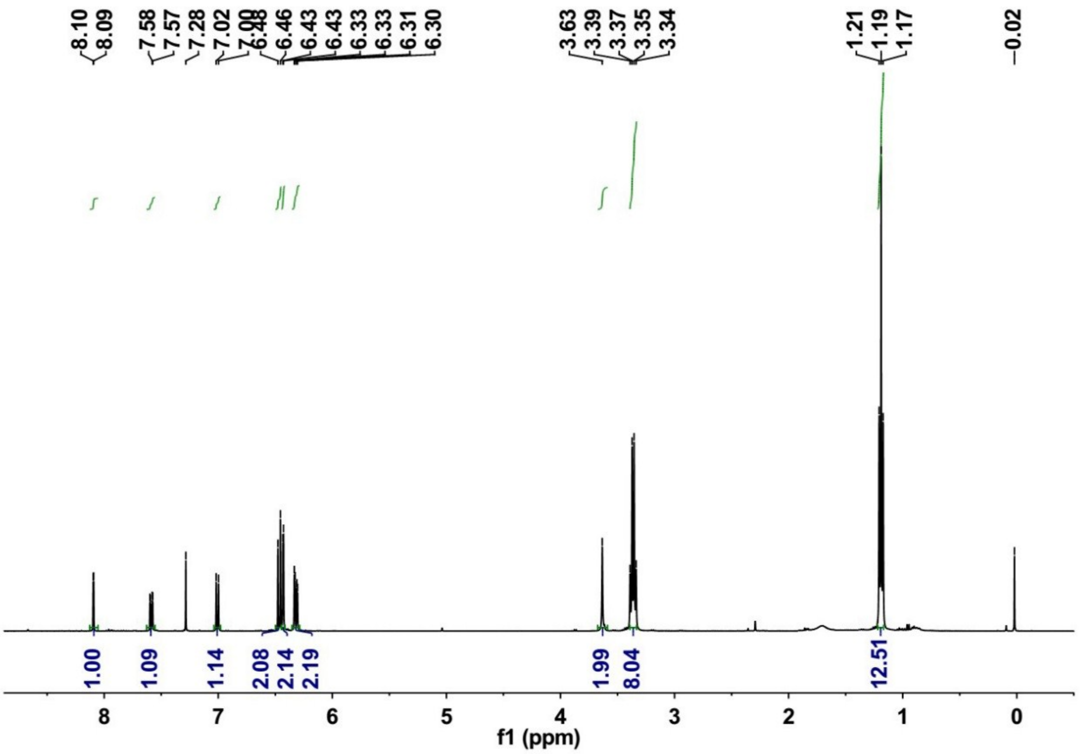

Figure S1. ^{1}H NMR spectrum of *m*-Br-RHZ in $CDCl_3$.

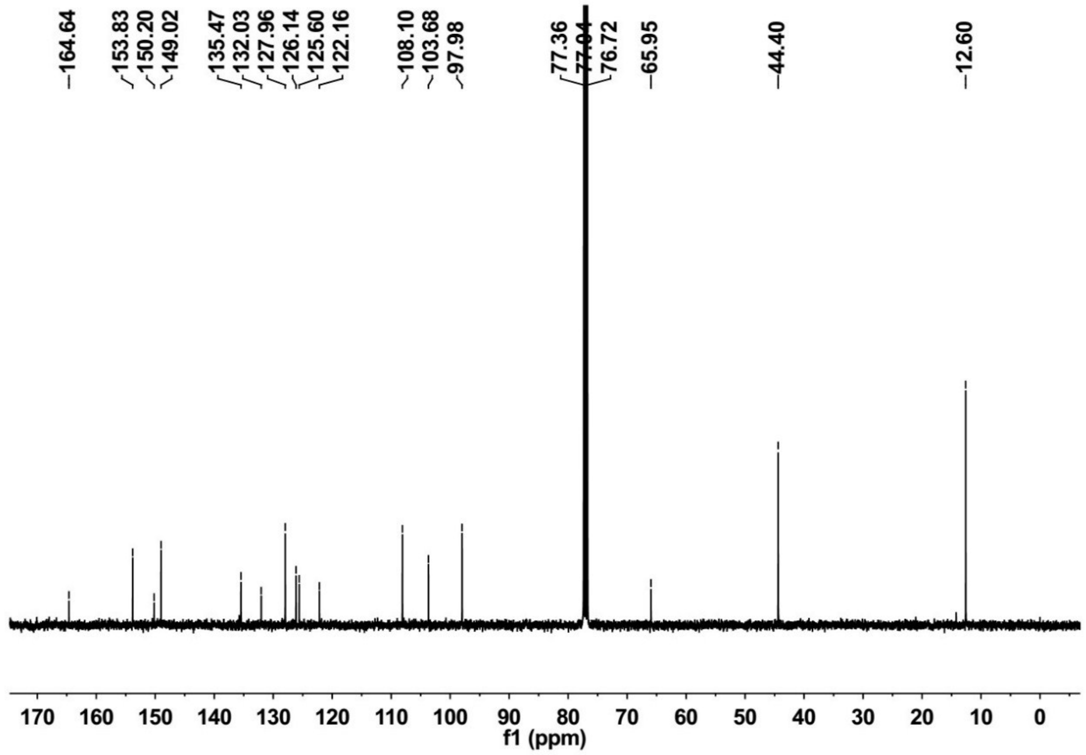

Figure S2. ^{13}C NMR spectrum of *m*-Br-RHZ in $CDCl_3$.

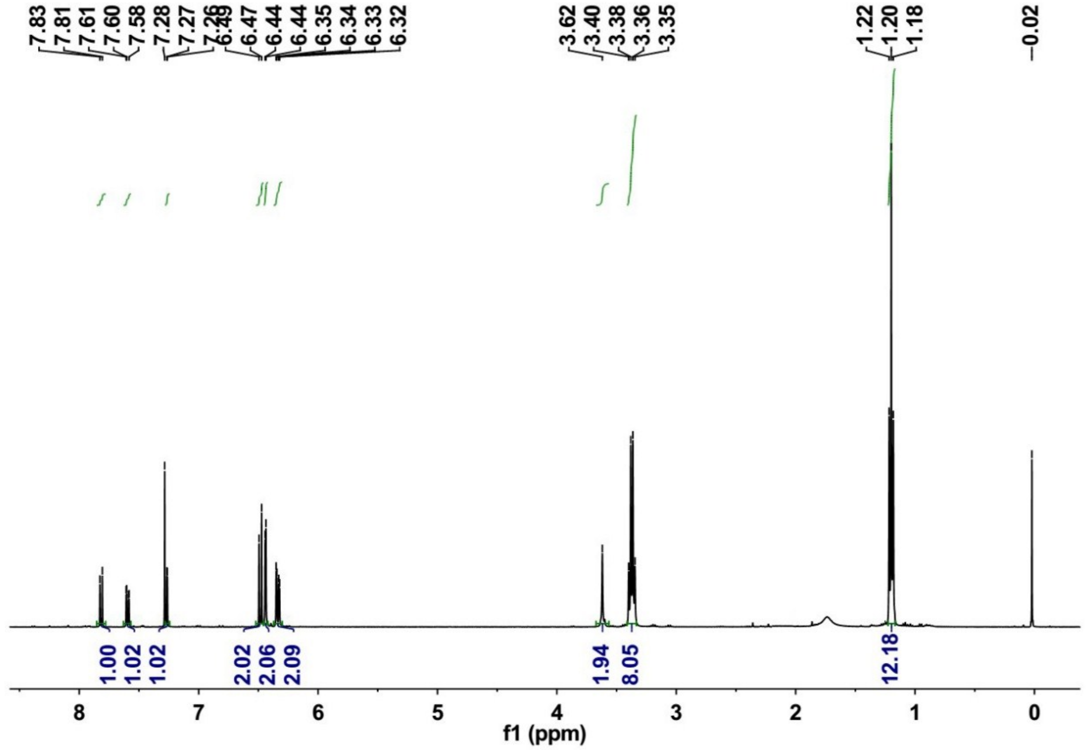

Figure S3. ^{1}H NMR spectrum of ***p*-Br-RHZ** in $CDCl_3$.

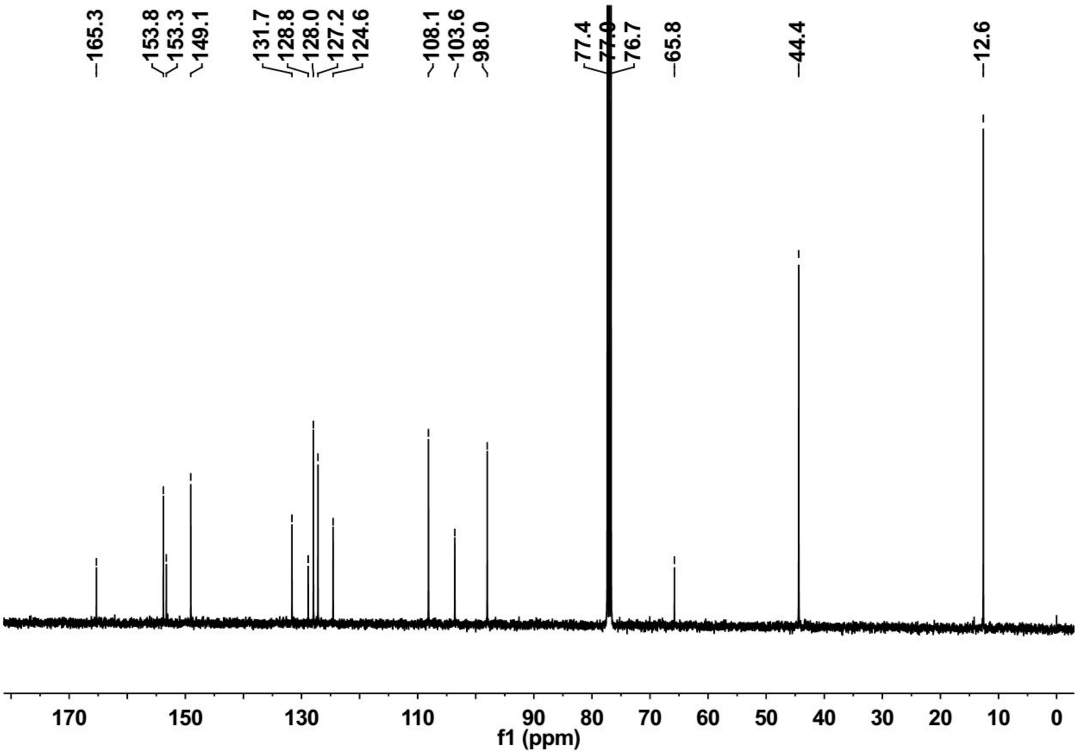

Figure S4. ^{13}C NMR spectrum of *p*-Br-RHZ in $CDCl_3$.

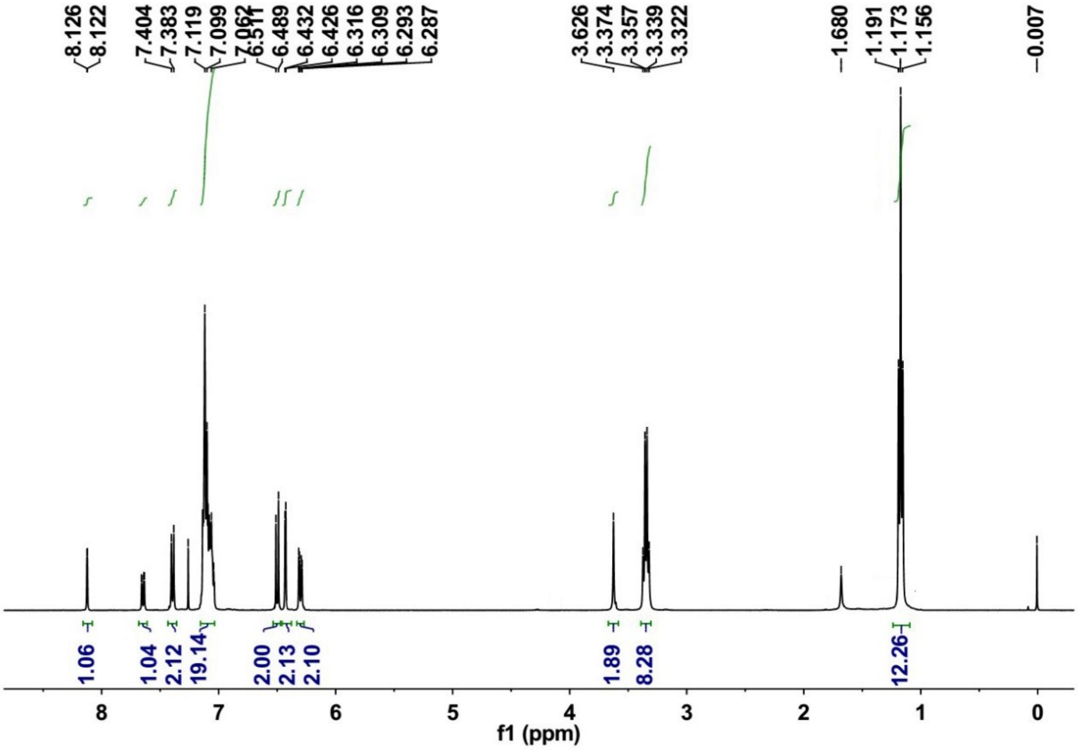

Figure S5. ^{1}H NMR spectrum of *m*-TPE-RHZ in $CDCl_3$.

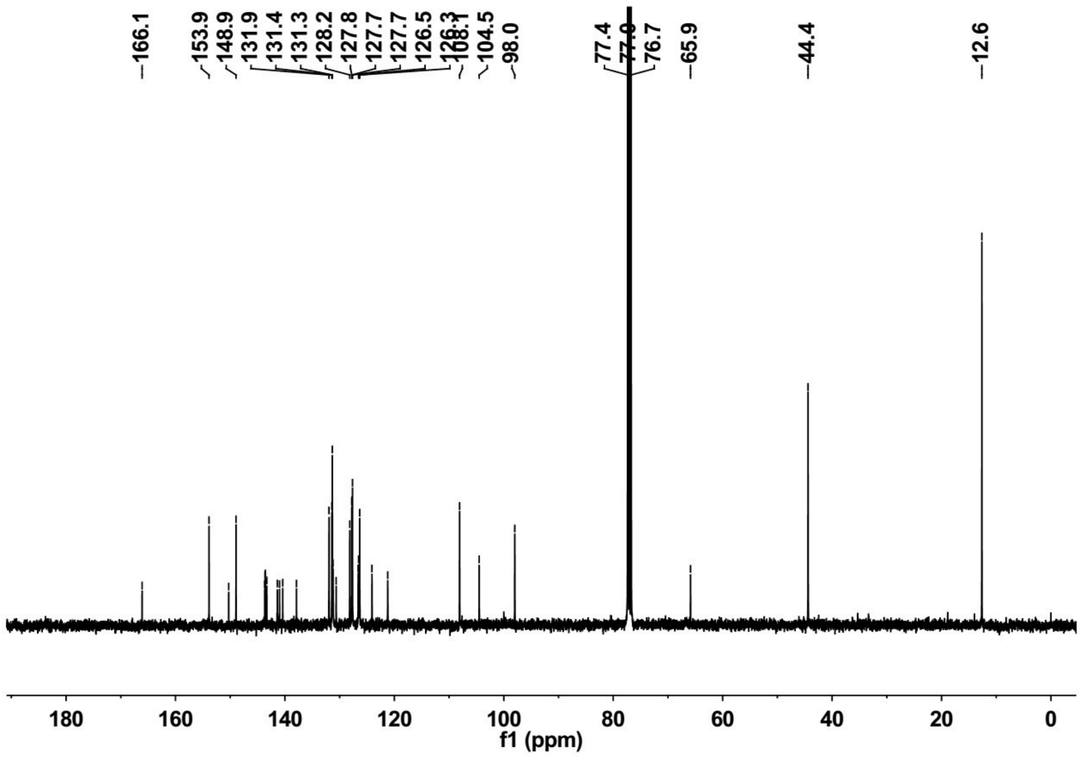

Figure S6. ^{13}C NMR spectrum of *m*-TPE-RHZ in $CDCl_3$.

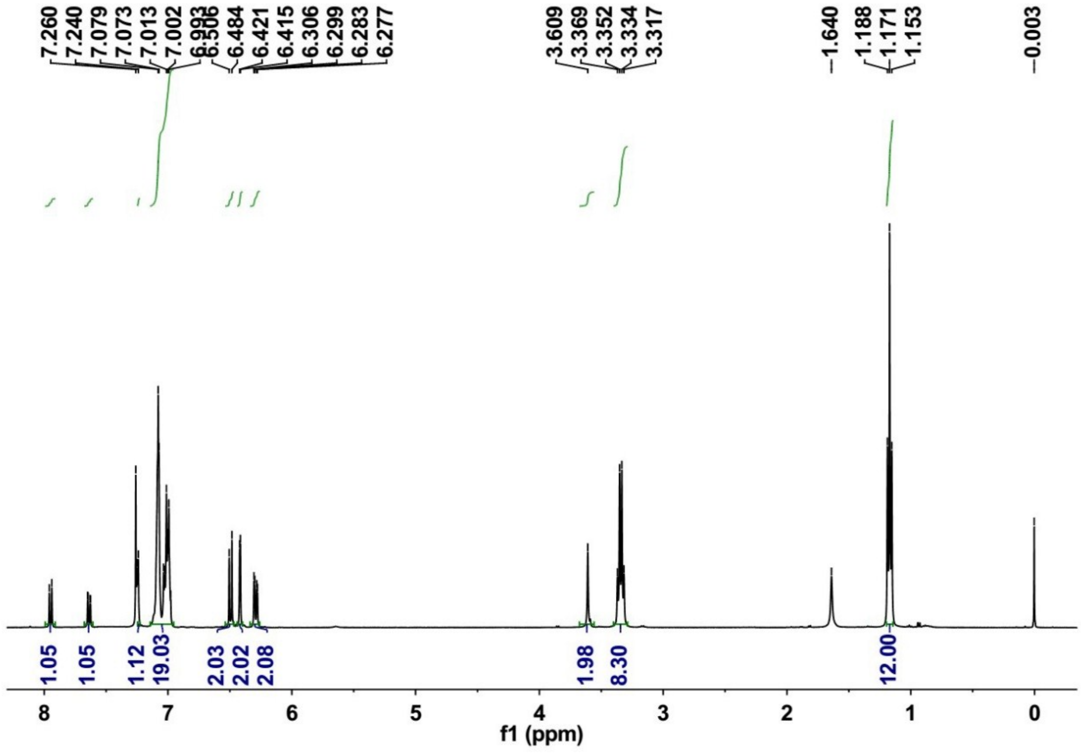

Figure S7. ^{1}H NMR spectrum of *p*-TPE-RHZ in $CDCl_3$.

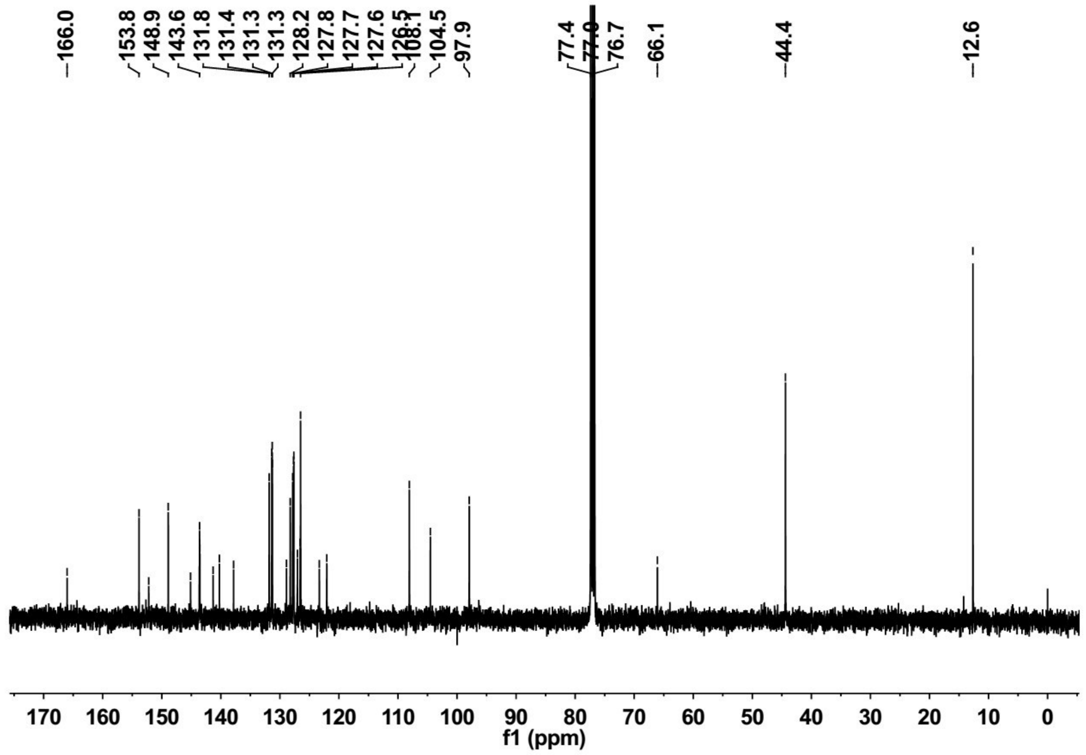

Figure S8. ^{13}C NMR spectrum of *p*-TPE-RHZ in $CDCl_3$.

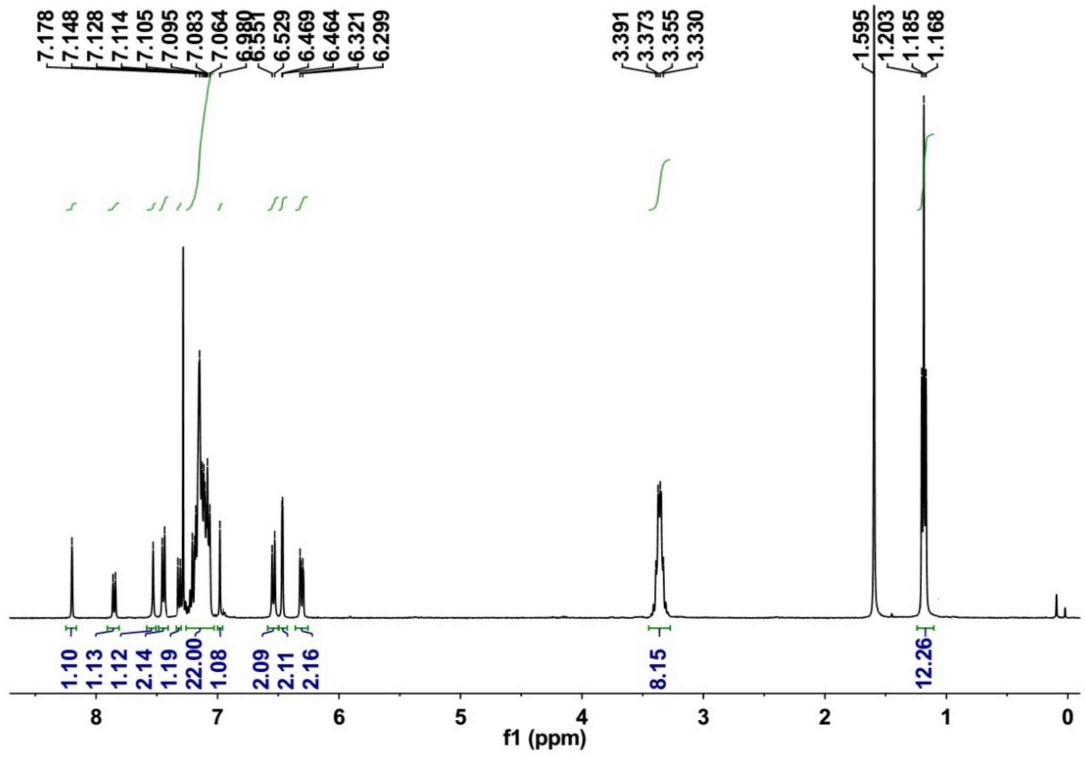

Figure S9. ^{1}H NMR spectrum of *m*-TPE-RNS in $CDCl_3$.

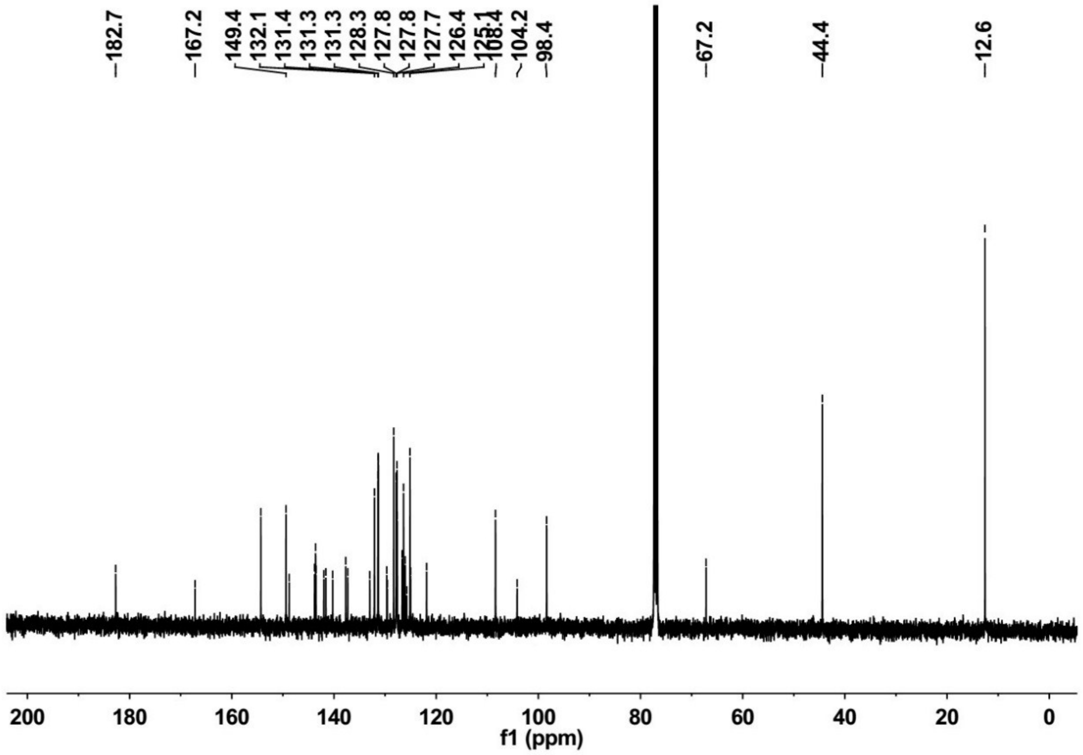

Figure S10. ^{13}C NMR spectrum of *m*-TPE-RNS in $CDCl_3$.

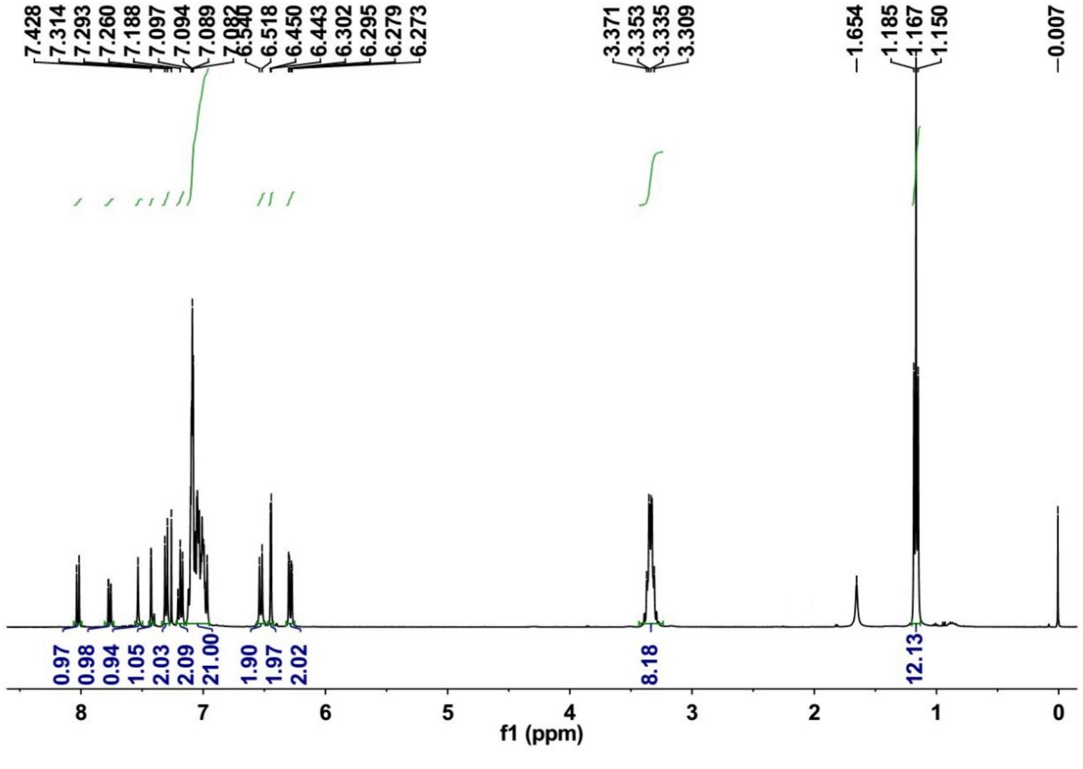

Figure S11. ^{1}H NMR spectrum of *p*-TPE-RNS in $CDCl_3$.

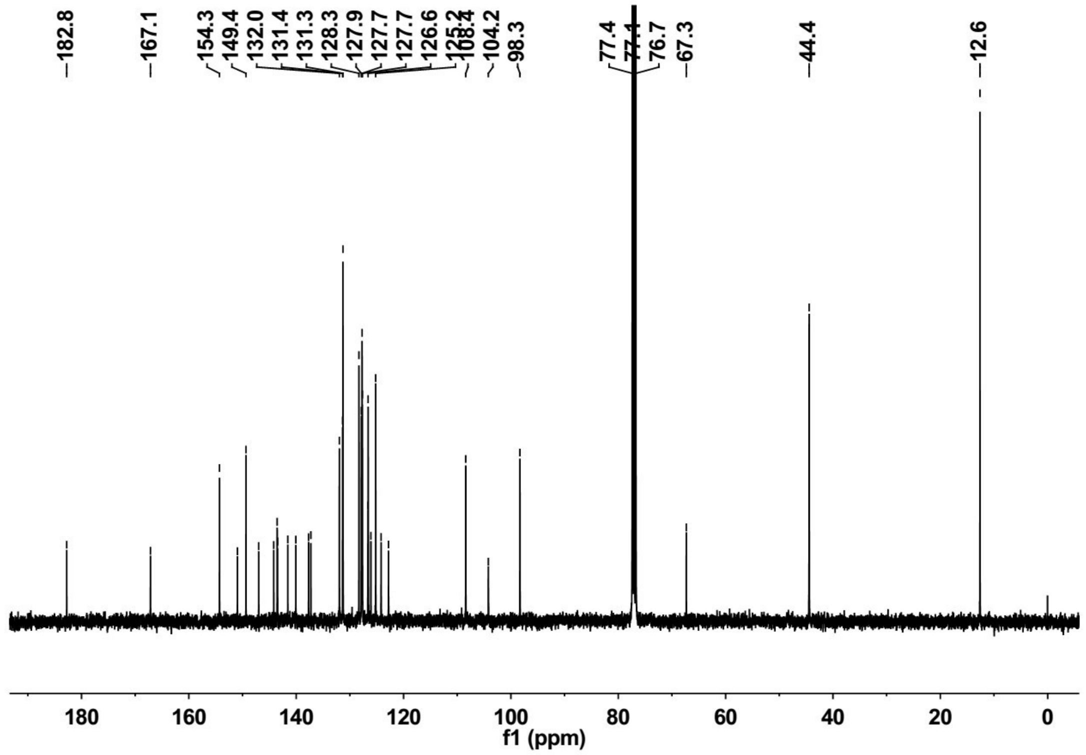

Figure S12. ^{13}C NMR spectrum of *p*-TPE-RNS in $CDCl_3$.

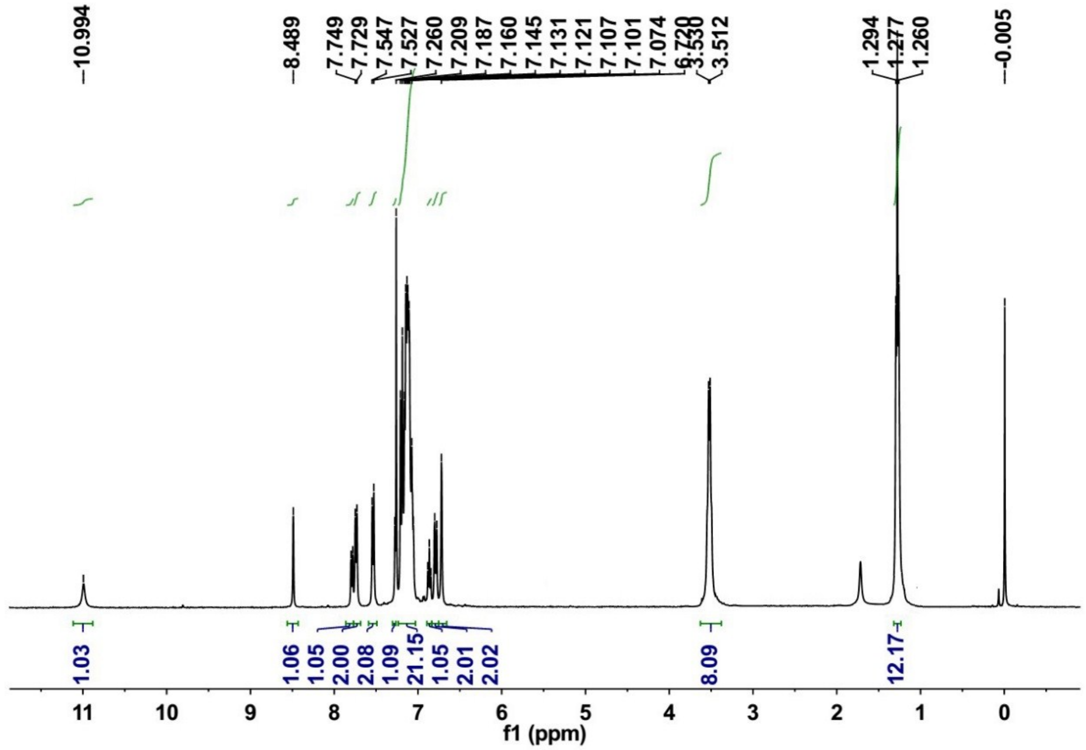

Figure S13. ^{1}H NMR spectrum of *m*-TPE-RNO in $CDCl_3$.

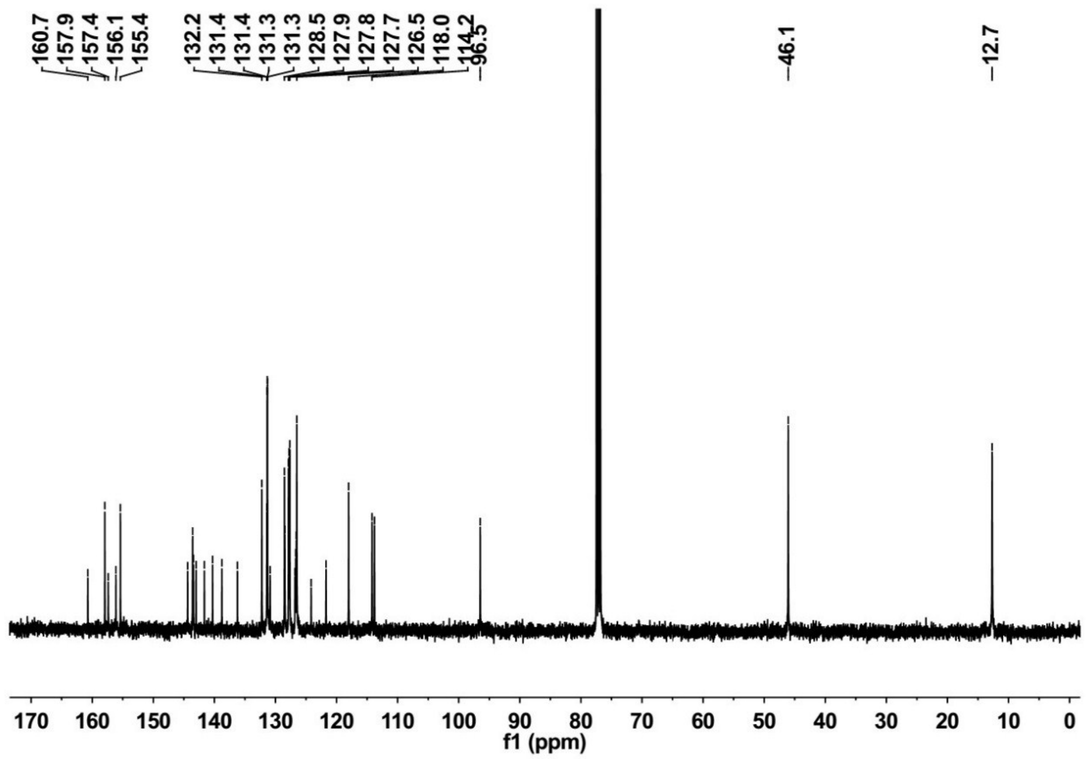

Figure S14. ^{13}C NMR spectrum of *m*-TPE-RNO in $CDCl_3$.

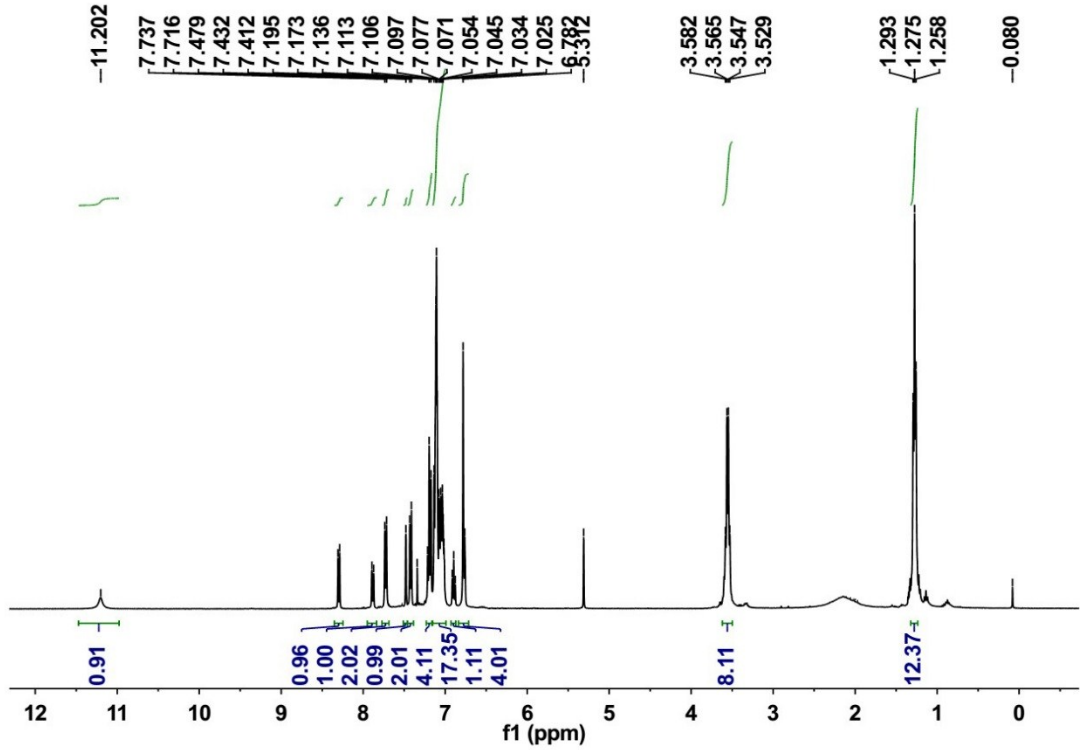

Figure S15. ^{1}H NMR spectrum of *p*-TPE-RNO in CD_2Cl_2.

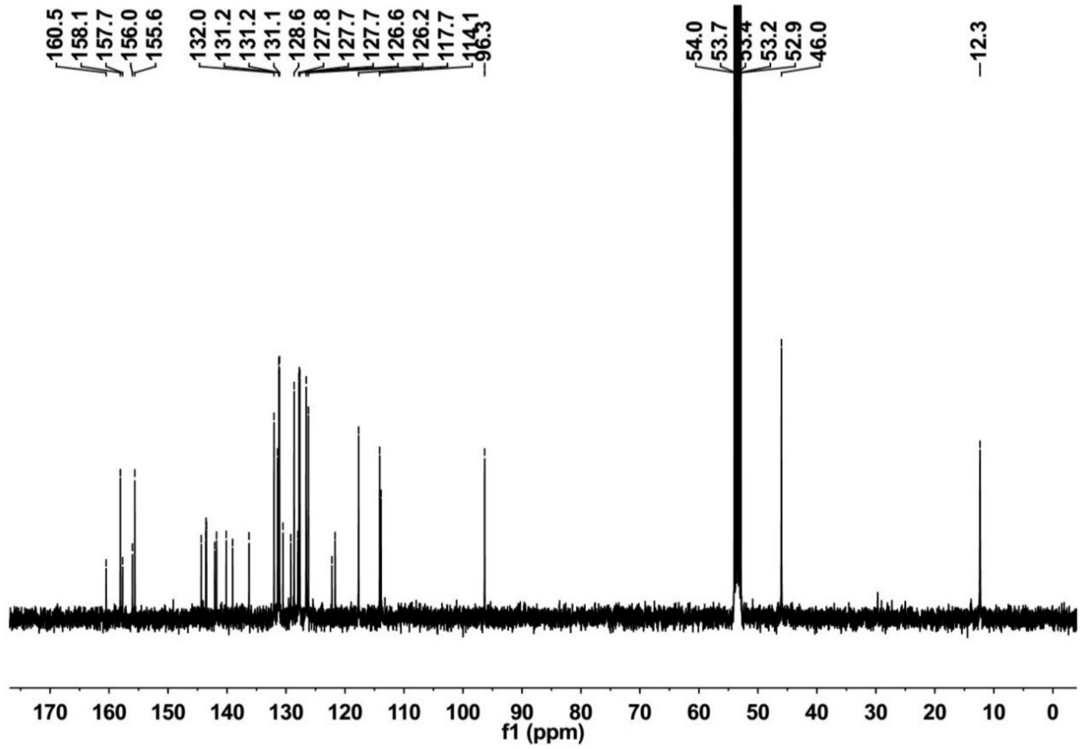

Figure S16. ^{13}C NMR spectrum of *p*-TPE-RNO in CD_2Cl_2.

2. Theoretical Calculations

Table S1. Selected parameters for the vertical excitation (UV-vis absorptions) of the named compounds Calculated by TDDFT//B3LYP/6-31G(d). (THF was employed as solvent in all the calculations). H stands for HOMO and L stands for LUMO, contributions were calculated from the CI coefficients of the wave functions for each excitations.

Compounds	Electronic Transition	Energy	Oscillator Strength (f)	Composition	Contribution
RNO	$S_0 \rightarrow S_1$	2.347 eV	0.0256	H → L	48.4%
				H-1 → L	51.6%
	$S_0 \rightarrow S_2$	2.615 eV	0.9941	H → L	52.0%
				H-1 → L	48.0%
p-TPE-RNO	$S_0 \rightarrow S_1$	2.093 eV	0.0017	H → L	92.7%
	$S_0 \rightarrow S_2$	2.377 eV	0.0190	H-1 → L	52.6%
				H-2 → L	39.9%
	$S_0 \rightarrow S_3$	2.613 eV	0.9558	H-1 → L	44.0%
				H-2 → L	56.0%
	$S_0 \rightarrow S_4$	3.035 eV	0.8363	H → L+1	96.7%
m-TPE-RNO	$S_0 \rightarrow S_1$	2.198 eV	0.0296	H → L	97.7%
	$S_0 \rightarrow S_2$	2.360 eV	0.0240	H-1 → L	43.4%
				H-2 → L	52.7%
	$S_0 \rightarrow S_3$	2.612 eV	0.9649	H-1 → L	55.0%
				H-2 → L	45.0%
	$S_0 \rightarrow S_6$	3.241 eV	0.4584	H → L+1	92.6%

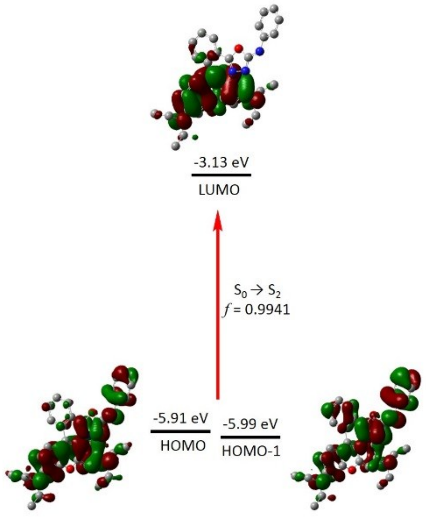

Figure S17. Time dependent B3LYP/6-31G(d) calculated frontier molecular orbitals, energy levels, allowed electronic transition (S_0-S_2) and oscillator strength of RNO. Hydrogen atoms are omitted for clarity.

3. AIE Curves

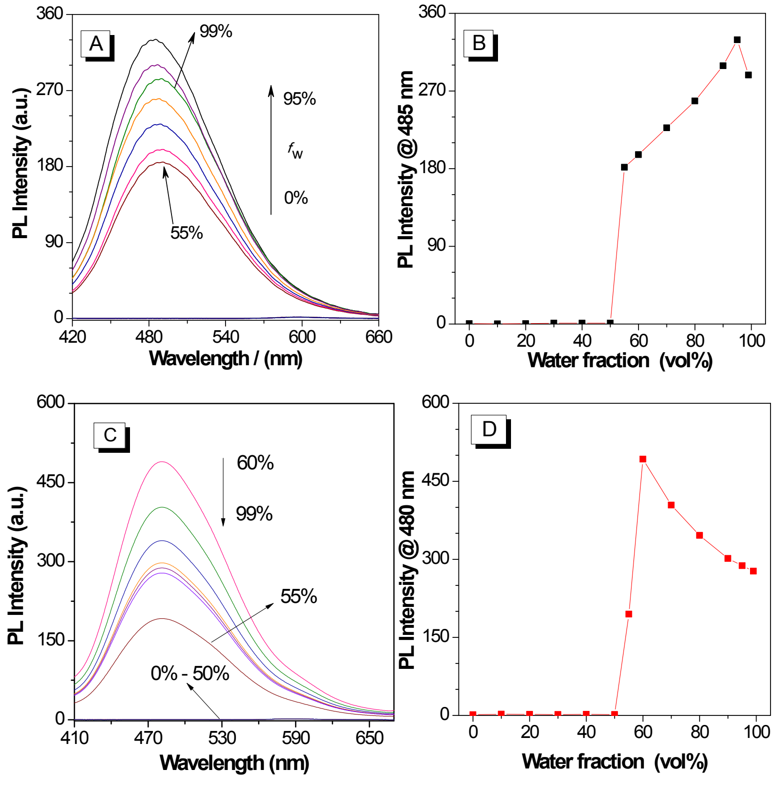

Figure S18. PL spectra of 10 µM *p*-TPE-RNS (A) and 10 µM *m*-TPE-RNS (C) in CH_3CN-water mixture with different water fraction; PL intensity profiles of 10 µM *p*-TPE-RNS (B) and 10 µM *m*-TPE-RNS (D) in CH_3CN-water mixture with different water fraction at 485 nm and 480 nm, respectively. Excitation, 355 nm.

4. Particle Size.

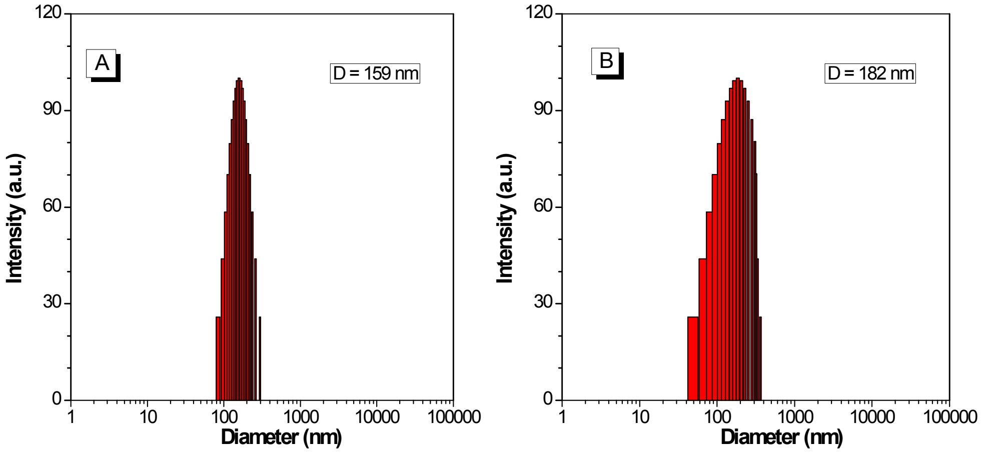

Figure S19. Particle sizes of the nano-aggregates of 10 μM *m*-TPE-RNS (A) and 10 μM *p*-TPE-RNS (B) in CH_3CN-water mixture with 60% water fraction measured by DLS.

5. AIE Property and Response to Hg^{2+}

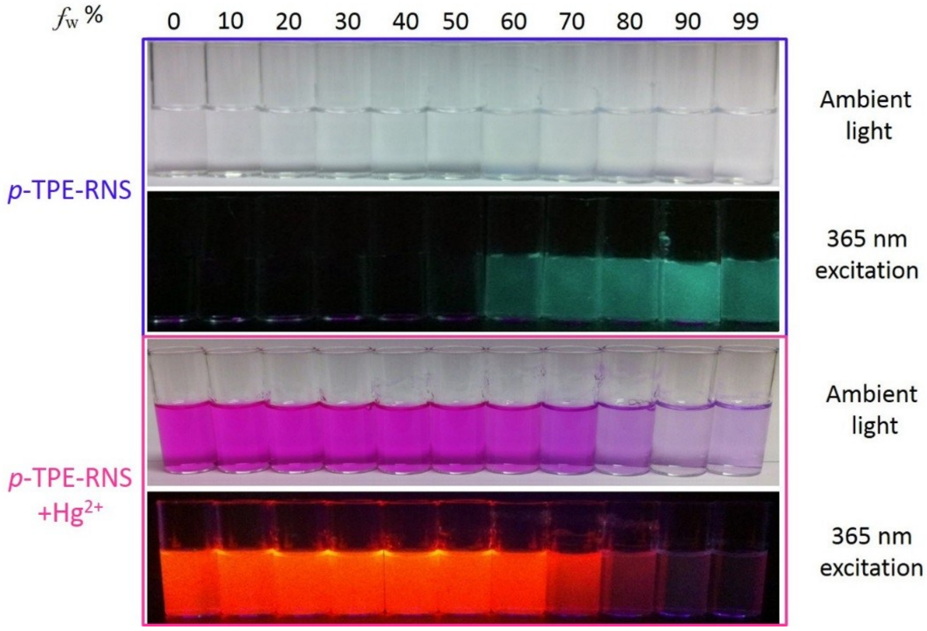

Figure S20. Photos of 10 μM *p*-TPE-RNS in CH_3CN-water mixture with different water fraction in the absence (row 1, row 2) and presence (row 3, row 4) of 2 equiv. of Hg^{2+}. Row1, row 3: under ambient light; row2, row 4: under excitation of a UV lamp at 365 nm.

6. Detect limit

The spectra of free sensors (0.1 μM) in CH_3CN:H_2O (3:7, v/v) mixture were collected for 10 times to determine the background noise σ. Then the solution was treated with various concentration of Hg^{2+} from 0-10.0 ppb, all fluorescence spectra were collected after mixing for 30 s. Linear regression curves was then fitted according to the data in the range of [Hg^{2+}] from 0 to 3.0 ppb, and the slopes of the curves was obtained (Figure S21). The detection limits (3σ /slope) were then determined to be 0.3 ppb and 1.2 ppb for *m*-TPE-RNS and *p*-TPE-RNS, respectively.

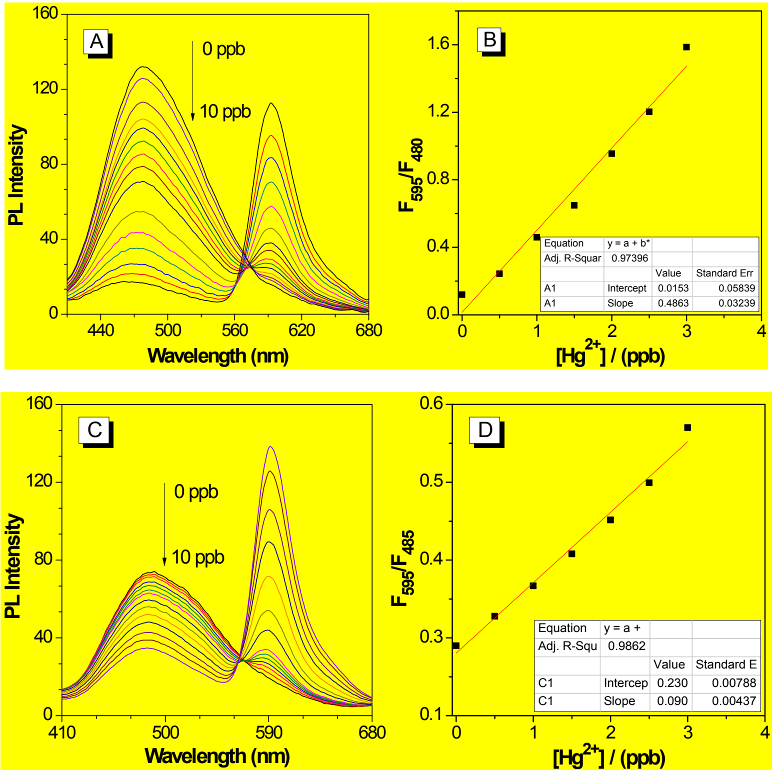

Figure S21. Fluorescent spectra of 0.1 μM *m*-TPE-RNS (A) and *p*-TPE-RNS (C) in CH_3CN-water mixture with 70% water fraction in the presence of 0-10 ppb of Hg^{2+}. The linear fit plots of 0.1 μM *m*-TPE-RNS (B) and *p*-TPE-RNS (D) in the range of 0-3 ppb of Hg^{2+}.

7. Cell Viability Test

Cells were seeded in 96-well plates at density of 5000 cells/well. After overnight culture, medium in each wells were replaced by fresh medium containing different concentrations of *p*-TPE-RNS. After 24 hours treatment, into each well, 10 μL MTT solution (5 mg/mL in phosphate buffer solution) was added. After 4 hours incubation at 37 °C, 100 μL SDS-HCl solution (10% SDS and 0.01 M HCl) was added to each well. After 6 hours incubation at 37 °C, the absorbance of each wells at 570 nm was recorded by the plate reader (Perkin-Elmer Victor3™).

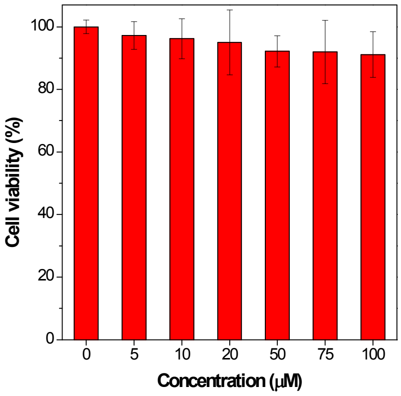

Figure S22. Cytotoxicity of *p*-TPE-RNS evaluated on HeLa cells by MTT assay.

References:

i. (a) L. Zhou, X. Zhang, Q. Wang, Y. Lv, G. Mao, A. Luo, Y. Wu, Y. Wu, J. Zhang and W. Tan, *J. Am. Chem. Soc.*, 2014, **136**, 9838; (b) J. Fan, P. Zhan, M. Hu, W. Sun, J. Tang, J. Wang, S. Sun, F. Song and X. Peng, *Org. Lett.*,

2013, **15**, 492.
